# Azobenzene as an Effective Ligand in Europium Chemistry—A Synthetic and Theoretical Study

**DOI:** 10.3390/molecules29215187

**Published:** 2024-11-02

**Authors:** Damian G. Allis, Ana Torvisco, Cody C. Webb, Miriam M. Gillett-Kunnath, Karin Ruhlandt-Senge

**Affiliations:** 1 Department of Chemistry, Syracuse University, Syracuse, NY 13210, USA; 2 Institute of Inorganic Chemistry, Technical University of Graz, 8010 Graz, Austria; 3 Department of Chemistry, SUNY Oneonta, Oneonta, NY 13820, USA; 4 Department for Physical & Environmental Sciences, University of Toronto, Scarborough, ON M1C 1A4, Canada

**Keywords:** europium, azobenzene, coordination chemistry, europium-π bonding, structural characterization

## Abstract

The preparation and characterization of two novel europium–azobenzene complexes that demonstrate the effectiveness of this ligand for stabilizing reactive, redox-active metals are reported. With the family of rare earth metals receiving attention due to their potential as catalysts, critical components in electronic devices, and, more recently, in biomedical applications, a detailed understanding of factors contributing to their coordination chemistry is of great importance for customizing their stability and reactivity. This study introduces azobenzene as an effective nonprotic ligand system that provides novel insights into rare earth metal coordination preferences, including factors contributing to the coordinative saturation of the large, divalent europium centers. The two compounds demonstrate the impact of the solvent donors (tetrahydrofuran (THF) and dimethoxyethane (DME)) on the overall coordination chemistry of the target compounds. Apart from the side-on coordination of the doubly-reduced azobenzene and the anticipated N-N bond elongation due to decreased bond order, the two compounds demonstrate the propensity of the europium centers towards limited metal-π interactions. The target compounds are available by direct metallation in a straightforward manner with good yields and purity. The compounds demonstrate the utility of the azobenzene ligands, which may function as singly- or doubly-reduced entities in conjunction with redox-active metals. An initial exploration into the computational modeling of these and similar complexes for subsequent property prediction and optimization is performed through a methodological survey of structure reproduction using density functional theory.

## 1. Introduction

The rare earth metals, or lanthanide elements (Ln), display a diverse structural chemistry as a consequence of their 4f-orbitals and ability to adopt multiple oxidation states [1]. The rare earth metals also display large ionic sizes, enabling insights into the factors responsible for stabilizing large metal centers [2]. Coordinative chemistry studies have shown rare earth compounds to adopt coordination numbers from six to twelve [2]. As a result, the variety of coordination chemistry among the rare earth metals potentially enables a wide range of functionality, with a detailed understanding of their coordinative preferences being a requisite for their use in various applications, including within a wealth of technically relevant materials [3,4]. With a goal to prepare tailored compounds for specific applications, studies focused on ligand design, co-ligand inclusion, and the ability of a ligand or co-ligand to support non-covalent interactions offer key insights. Further, understanding a ligand’s ability to support compounds in a range of oxidation states is essential.

It has been well documented that structural and chemical similarities exist between rare earth metals capable of adopting oxidation state +2 and the heavy alkaline earth (Ae) metals, calcium and strontium [5,6]. Divalent europium and samarium are almost identical in size to the strontium ion, and divalent ytterbium closely resembles the calcium ion. However, the largest alkaline earth metal, barium, has no rare earth equivalent (Table 1). Moreover, the redox potentials of the metal pairings compare favorably. These similarities in periodic properties have informed our synthetic approach to working with Ln systems.

For both families of compounds, extensive studies are available examining factors responsible for the steric stabilization of these large, highly reactive metal centers. Several studies have shown the impact of non-covalent, secondary interactions as an effective means of providing additional steric stabilization, yielding effective precursors for technical applications. Secondary interactions specifically include metal-π and metal-F interactions, as well as agostic interactions [10,11]. Hydrogen bonding, which is also considered a structure-determining non-covalent interaction [12], is not considered in this study, as the presence of protic moieties results in the protolysis of the target compounds. In contrast to the well-demonstrated impact of secondary interactions in describing the behavior of both rare earth and heavy alkaline earth compounds, relatively little systematic work exists to examine the impact of those interactions. With the aim of better understanding the coordination chemistry and impact of the non-covalent interactions for technical applications, it is our goal to develop the means to rationally design ideal precursors.

Secondary interactions are especially important in compounds possessing large ionic radii and weak metal–ligand bonds. As non-covalent interactions are relatively weak, they play only a minor role in transition metal chemistry, where stronger metal-ligand bonds are observed. However, the large rare earth or alkaline earth metals provide the critical steric saturation at the metal centers to suppress aggregation, rendering the compounds more volatile and soluble. An impressive example of the impact of secondary interactions is in the use of the -OC(CF_3_)_3_ ligand and resulting alkaline earth-fluorine interactions that have afforded families of mono- and heterobimetallic alkali/alkaline earth compounds with superior thermal properties and volatility [13,14]. Aside from metal-F interactions, recent work in our group and others has demonstrated the utility of metal-π interactions for stabilizing large, highly reactive metal centers. Examples include a series of heterobimetallic Ae-Ln(II) complexes using the ligand 2,6-diphenylphenolate (Odpp) [6]. These complexes differ in their association modes (polymeric vs. charge-separated species) while exhibiting varying ranges of hapticity [5,6,15,16].

This paper focuses on europium, a metal of great interest, and several recent reports focus on europium species for optoelectronic, biomedical, and anticounterfeiting applications [17,18,19]. The ability to prepare rare earth species with tailored properties further enhances their utility, as optimized precursors extend the compounds’ use in various applications. Rare earth compounds have a well-documented history of use, as summarized in a comprehensive review article [1].

**Figure 1:**
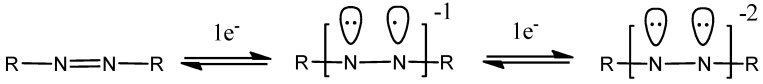
Modes of the azobenzene ligand (R = C_6_H_5_) in order of decreasing bond order (from left to right: 2, 1.5, 1).

We have chosen the azobenzene (N_2_Ph_2_) ligand as a redox-active, aprotic ligand that offers versatile binding and bridging modes when reduced. Further, the N-N moiety can potentially bind in several ways to the metal centers, with the phenyl groups also capable of additional coordinative stabilization via metal-π interactions. Upon reduction, the N-N double bond is initially converted to a monoanionic radical and then a dianionic species, as shown in Figure 1. The N-N bond length increases in accord with the reduction in bond order, increasing from 1.24 Å for the double bond to 1.41Å for the dianionic species [20].

Azobenzene exhibits a bright orange color in its neutral state and has conventionally been used in organic dyes but has since found biochemical uses as a chromophore [21,22]. Due to their luminescent properties, europium coordination complexes are of primary interest for use in bioassays and cellular optical imaging. Despite its great potential as a ligand, surprisingly few examples of azobenzene compounds have appeared in the literature [23,24,25]. Azobenzene primarily exists as the anion (PhNNPh)2−for divalent Ln metals, as reported in a few select publications focusing on samarium [26,27,28]. One example, the complex [SmI(thf)_3_]_2_(µ-η^2^:η^2^-N_2_), provides confirmation of the dianionic state of azobenzene with the reported N-N bond length of 1.467(6) Å [28]. Additionally, a few examples have focused on dinitrogen as a ligand [29].

We here present the first europium azobenzene compounds, namely the tetrahydrofuran (THF) adduct [Eu(thf)_3_]_2_(µ-η^1^:η^1^-N_2_Ph_2_)_2_ and the dimethoxyethane (DME) analogue [Eu(dme)_2_]_2_(µ-η^2^:η^2^N_2_Ph_2_)_2_.

## 2. Results and Discussion

### 2.1. Synthesis

**Scheme 1:**
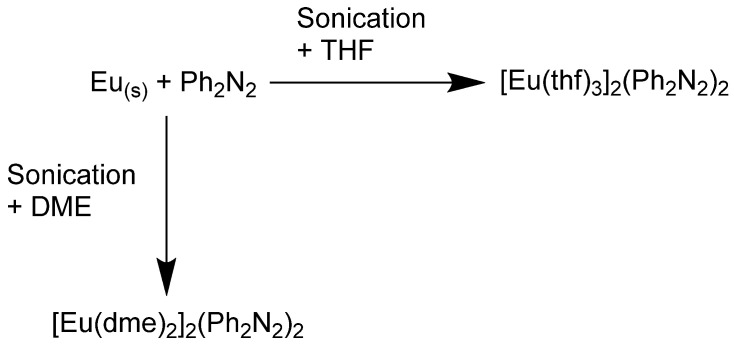
Synthesis of Eu-azobenzene complexes by direct metallation.

The target compounds were synthesized by direct metallation (Scheme 1), resulting in [Eu(thf)_3_]_2_(µ-η^1^:η^1^-N_2_Ph_2_)_2_, **1**, and [Eu(dme)_2_]_2_(µ-η^2^:η^2^N_2_Ph_2_)_2_, **2**. Europium metal and azobenzene (Ph_2_N_2_) were combined in equimolar amounts in a reaction vessel under inert gas and suspended in tetrahydrofuran (THF, for compound **1**) or 1,2-dimethoxyethane (DME, for compound **2**), as represented in Scheme 1.

Filing the Eu metal into fine pieces was sufficient to achieve metal activation. The reaction vessels were sonicated for four days and then slowly cooled, during which pure compounds and the formation of X-ray quality crystals were afforded (compound **1**: 44% yield, compounds **2**: 67% yield, unoptimized) in good purity, as confirmed by ^1^H NMR spectroscopy (Supporting Information Figures S1 and S2). NMR studies also confirmed the 2:2 ligand:donor ratio observed in the crystallographic study.

### 2.2. X-Ray Crystal Structures

**Figure 2:**
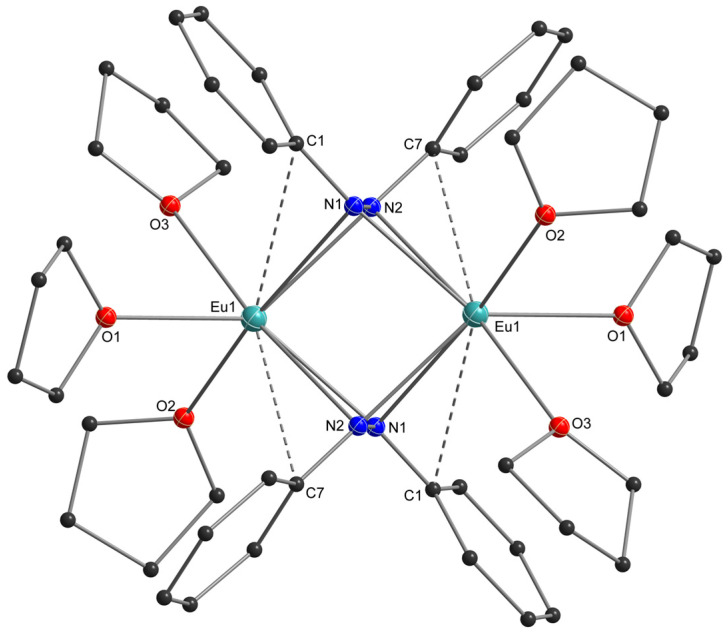
Crystal structure of compound **1**. Hydrogen atoms have been omitted for clarity. The additional stabilization of the europium centers through Eu-π coordination to the ligand phenyl rings is shown as a dotted line.

Compound **1**, shown in Figure 2, crystallizes as a dimeric complex in the monoclinic space group P2_1_/n, with two 1,2-diphenylhydrazido(2-) ligands bridging the two europium centers. The complex crystallizes as a seven-coordinate THF-solvate. As the N-Eu-N angles are very narrow (32.93° and 25.57°), we consider these interactions from a coordinative chemistry point of view as a single point of attachment, with one of these contacts arising from each N_2_Ph_2_ ligand, resulting in a formally five-coordinate species. Each metal center also exhibits two metal-π interactions stemming from the ipso-carbon atoms on the phenyl rings attached to the azo moieties. The Eu···C1 interaction exhibits a distance of 3.19 Å while the Eu···C7 interaction is slightly longer at 3.28 Å. These values are consistent with the reported cutoff range (2.98–3.29 Å) for Eu···Cπ interactions, but the latter values can be considered very weak [6].

**Figure 3:**
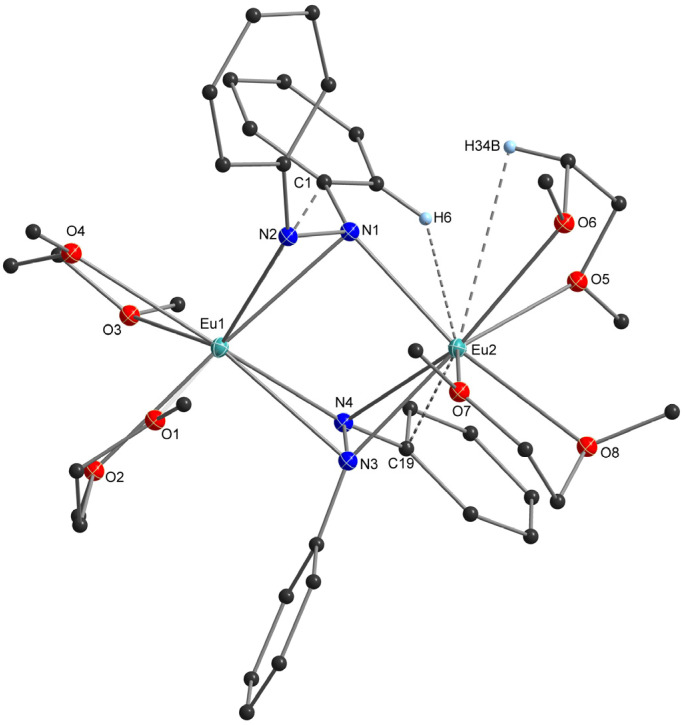
The crystal structure of compound **2**. Hydrogen atoms, except those involved in agostic interactions, have been omitted for clarity. The additional stabilization of the europium centers through Eu-π coordination to the ligand phenyl rings are shown as dotted lines. All hydrogen atoms have been placed in calculated positions using a riding model.

Compound **2**, shown in Figure 3, crystallizes as a dimeric complex in the P-1 triclinic space group, with two DME molecules coordinating to each of the two europium centers. Two 1,2-diphenylhydrazido(2-) ligands asymmetrically bridge the metal centers with Eu2 only receiving a single nitrogen contact point from one of the dianionic ligands, while the second azobenzene ligand coordinates to the metal via both nitrogen atoms, similar to the symmetric N-Eu-N bonding mode seen in compound **1**. Eu1 receives a single Eu···C(π) interaction (3.09 Å), providing an overall 6 + 1π coordination pattern. Eu2 also receives a single C(π) interaction (3.13 Å), but it has two additional agostic Eu···H-C contacts. The first agostic interaction coordinates from H6 (3.25 Å) and the second coordinates from a DME molecule (3.21 Å), giving Eu2 an overall coordination of 6 + 1π + 2M^…^H. It is notable that additional secondary non-covalent interactions compensate for the reduced metal-nitrogen coordination. The Supporting Information (Table S1) summarizes the crystallographic information, data collection, and structure refinement parameters.

## 3. Discussion

### 3.1. Synthetic and Spectral Aspects

The target compounds were synthesized by direct metalation. Filing the metal is crucial to increase surface area and facilitate the reaction with azobenzene. Reactivity was further promoted by activating the metal under ultrasonic conditions. Alternative routes to preparing the target compounds were attempted by dissolving europium in anhydrous liquid ammonia under the addition of azobenzene and the appropriate co-ligand, but single crystals could not be isolated from the resulting precipitates. The low redox potential of Eu required more stringent conditions, with a reaction time of four days to the initial formation of observable crystals.

Single-crystal X-ray crystallography and infrared (IR) spectroscopy results confirm the dianionic charge and reduction of bond order of azobenzene based on the N-N bond length. In compound **1**, both azobenzene ligands coordinate to Eu, exhibiting an N-N bond length of 1.471(3) Å, while those in compound **2** are observed at 1.472(8) Å, consistent with reported N-N distances for reduced azobenzene [20]. In the IR spectra, the N=N stretching frequency for the neutral ligand is observed at 1450 cm^−1^, while the reduced N-N single bond stretch occurs in the range of 1030–1110 cm^−1^. Our results confirm the presence of only the N-N single bond stretching frequency observed for **1** and **2** in the IR spectrum at 1071 cm^−1^ with no stretching bands indicative of N=N frequencies.

^1^H-NMR analysis was also performed to support our crystal structure determinations and to assay bulk purity of compounds **1** and **2**, which exhibit characteristic peaks for the phenyl groups on azobenzene, with the *ortho*-protons positioned furthest downfield. The *o*-C_6_H_6_ signals, typically found near 8.0 ppm in the ^1^H-NMR spectra (Supporting Information Figures S1 and S2) were used as a reference for integrating the rest of the peaks in the spectrum. The *o*-C_6_H_6_ peak in our spectra exhibited an upfield shift of approximately 1 ppm, consistent with a reported ^1^H-NMR spectrum of diphenylhydrazine, which contains a single N-N bond similar to that of compounds **1** and **2**. Bulk purity was also analyzed based on ^1^H-NMR results by making use of the ratio of co-ligand (THF or DME) to ligand (azobenzene). This supports the assertion that the sample studied by XRD is representative of the bulk product used in NMR and IR spectral analyses.

### 3.2. Structural Aspects

Complexes **1** and **2** are both dimeric, doubly-bridged, THF and DME solvates of Eu containing the ligand 1,2-diphenylhydrazido(^2−^). The resulting acute N-Eu-N angles (Table 2) are sufficiently narrow that we can ascribe each bond as a single coordination from azobenzene to each Eu atom (Table 3).

For compound **1**, the bridging motif between each monomeric metal center results in a symmetric complex with two sets of Eu-N bond lengths, the longer of which are the Eu(1)-N(1) and Eu(1a)-N(2a) bonds (2.671 (2)–2.675 (2) Å), with Eu(1)-N(2) and Eu(1a)-N(1a) shorter at 2.456 (2)–2.469 (3) Å. The symmetric bridging motif is similar to the dimeric strontium pyrazolate [^t^Sr(^t^Bu_2_pz_2_(thf)]_2_ complex, which utilizes the 3,5-di-*tert*-butylpyrazolate ligand system that coordinates via a N-N contact in a fashion similar to azobenzene. In this strontium-based structure, the alkaline earth element most similar in ionic size to europium, two analogous sets of Sr-N bond lengths are observed, including shorter (2.473 (2)–2.514 (2)Å) and longer (2.786 (2)–2.792 (1) Å)) distances [15]. The slight elongation of the Sr-N bond lengths is likely due to the increased steric bulk of the three-dimensional tert-butyl groups on the pyrazolate ligand, as compared to the flat aryl moiety, which results in a reduced steric ligand bulk for the azobenzene. A reported complex, [{Eu(*t*Bu_2_Pz)_2_(THF)}_2_], exhibits an identical bridging pattern over the N-N moiety, further supporting the structure shown for compound **1**.

Compound **2** exhibits an asymmetric bridging motif where one azobenzene coordinates similarly to the pattern in compound **1**, while a second azobenzene has two contact points to one Eu center and only one contact to the other Eu center. There are two sets of Eu1-N bond lengths: the shorter set consisting of Eu(1)-N(2) and Eu(1)-N(4) (2.495 (5)–2.499 (5) Å) and the longer bond distances from Eu(1)-N(1) and Eu(1)-N(3) (2.708 (6)–2.644 (7) Å). This is further consistent with Eu-N bonds in compound **1**. The Eu(2)-N(1) and Eu(2)-N(3) bonds are similar in length (2.455 (7)–2.444 (6) Å), with one additional longer contact from Eu(2)-N(4) (2.730 (6) Å). There is no contact from Eu(2) to N(2). This asymmetrical μ-η^2^:η^1^ bridging pattern is rare but has been observed in a few dimeric complexes using pyrazolates containing dysprosium, molybdenium, or scandium [16,30]. One example, the rare earth [Dy(Ph_2_pz)_6_], exhibits an identical effect on the bond lengths where the symmetric bridging results in the shorter set of Dy-N contacts (2.457 (8)–2.417 (8) Å) while exhibiting an additional longer single contact to a bridging ligand (2.526 (8) Å) [31]. Notably, both metals in these previously reported complexes exhibit the three-contact bridging motif, while compound **2** has only one metal coordinated in this fashion.

The directions that can be taken to improve the chemical properties of any family of coordination complexes for a given application are limited only by resources and interest. Despite the differences in kind and number of coordinating ligands and number of resulting Eu-O interactions for **1** and **2**, both share a high degree of structural similarity in that both can be thought of as composed of an equatorial ring of azobenzene ligands (that define the pairing of the Eu centers) and “poles” of Eu-O-bound ligands. The clustering of phenyl groups to a central ring and saturated hydrocarbons to either pole is notable in the unit cell, where C-H^…^π interactions are prevalent between neighboring complexes by the “ring” of one complex interacting with the “pole” of its nearest neighbor.

### 3.3. Computational Modeling

The choice of solvating ligand reveals one level of property-tuning that retains the same basic coordination framework in both. Exploration of other means to chemically changing the behavior of complexes based on the structures of **1** and **2** by computational means is of interest in our group to more rapidly explore the possible tailoring of complexes for further property control. As a first step in this larger process, a method survey was performed comparing the crystal geometry of **1** to a selection of density functional/dispersion correction/basis set combinations to identify if any specific levels of theory were better suited to reproducing the structure of this complex. In the case of **1**, a small and relatively high symmetry complex is an excellent test structure for not only comparing theoretical methods against an experimental data set but also specifically for considering the gas-phase/crystal-phase differences in geometry that come from the various inter-complex interactions within the crystal.

With considerable precedent available for the framework C/H/N/O interatomic distances, focus here is placed on the quality of the treatment of the distances involving the symmetry-unique Eu atoms as compared to the experiment (those distances reported in Table 2 and Table 3). Bond length differences from the crystallographic structure for the Eu-Eu/N/O and Eu-C(π) interactions across all methods are provided in Table S3. Table S4 contains the RMSDs of the structures compared to the experiment (excluding H atoms), the diagnostic vibrational modes for the N=N stretches in each system, the NPA charges for the Eu and N atoms in each structure, and the time-per-SCF-cycle as a practical consideration of the compute times for each theory level on identical computers (for the consideration of the increases in time required to improve agreement).

**Figure 4:**
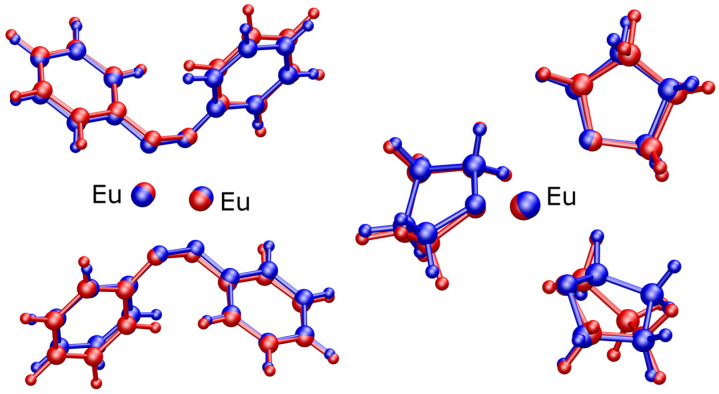
Position differences of the two azobenzene (left) and three THF (right) ligands for the crystal geometry (red) and the representative LC-ωHPBE-GD3(BJ)/Def2-SVP (blue) geometry. The largest apparent difference across all calculations is the change in a single THF orientation upon optimization across all methods (bottom-right).

From the large data set obtained within this computational survey for this one complex, several trends and assessments can be made:

- For **1,** across these ranges of theory, increasing the basis set size (and computational cost) from Def2-SVP to Def2-TVZ does not improve agreement with the crystal structure geometry except when using the LC-ωHPBE density functional with no dispersion correction.
- The LC-ωHPBE density functional with the GD3(BJ) dispersion correction produces geometries with RMSDs nearly half those of the remainder of the survey. We note that this does not mean that the LC-ωHPBE density functional is the means to producing the best gas-phase geometry until it can be determined that the geometry of the complex is only minimally impacted by crystal packing interactions. In the case of **1**, where inter-complex interactions are predominantly between THF C-H bonds and azobenzene phenyl rings, such a condition might be completely reasonable.
- Except for the Eu-N1 distance (one of the two longer Eu-N distances), the inclusion of dispersion corrections uniformly improves agreement with the experiment for all of the considered theory levels.
- NPA comparisons show a uniform sensitivity to the natural charges on the Eu atoms with a choice of SVP or TZV but only a fractional difference between N atoms in either case (and similar for the O atoms, which uniformly have predicted natural charges in the −0.55 to −0.59 e^−^ range).
- Considering the Eu-C(π) distances among the calculations reveals that using the smaller Def2-SVP basis set leads to markedly better agreement with the crystal geometry than Def2-TZV, and that dispersion corrections provide only fractional additional improvements in agreement. In terms of the generation of molecular structures for computational assessment, the crystal geometry of **1** can be better reproduced with theory levels differing only in the basis set that reduces the compute time per SCF cycle by 50% or more.
- With some small variation across all methods, the calculated Eu-C(π) distances are all in good agreement with the experiment and within the range of accepted values for these interactions. The removal of these complexes from the crystal environment and optimization as gas-phase species preserved these distances, indicating that their formation is not directly attributable to crystal packing but instead some energetic preference for including these coordinative interactions as part of the overall environment around each metal.
- Geometry comparisons (Figure 4) reveal that the shifting of the THF C_4_H_8_ chains during energy minimization produces the largest differences between gas-phase theory and crystal geometry. This is by no means unexpected, as the complete encapsulation of the Eu metals by the most electronegative atoms in these complexes leaves only the ligand fragments capable of the weakest of electrostatic interactions to undergo the structurally large but energetically small changes as part of crystal packing.
- There is clear disagreement in the prediction of the vibrational mode energy for the N=N stretch in the isolated *cis*-azobenzene using Def2-SVP vs. Def2-TZV, where Def2-SVP calculations can differ from the accepted 1511 cm^−1^ value by 11% to 20%. In the complex, however, Def2-SVP and Def2-TZV predicted mode energies for the symmetric and asymmetric N=N stretching pair differ by only a few cm^−1^ in all cases. Again, the time savings from using Def2-SVP across both optimization and normal mode analysis are considerable over Def2-TZV for the same density functionals and dispersion corrections.
- The one notable difference between LC-ωHPBE and the other three density functionals is to be found in the prediction that the N=N stretching modes are not localized to a single dominant stretching mode. With LC-ωHPBE, the lower-energy pair of stretches is coupled to phenyl ring expansion modes, while the higher-energy pair is coupled to phenyl lateral wagging modes. This splitting is 10 cm^−1^ with Def2-SVP and 40 cm^−1^ with Def2-TZV.

## 4. Materials and Methods

### 4.1. General Methods and Synthesis

All reactions were performed under inert conditions, which excluded air and water using a drybox, a solvent purification apparatus, and Schlenk techniques. IR spectra were collected using a Nicolet L200 FTIR spectrometer (Nicolet, Prague, Czech Republic) over the range of 4000–400 cm^−1^. IR samples were prepared using mineral oil mulls suspended between KBr plates. ^1^H NMR was collected using a 300 MHz Bruker Avance spectrometer (Bruker, Billerica, Massachusetts, USA) using D_6_-benzene (7.16 ppm). Melting points were measured on a Mel-temp II bench-top device, and samples were prepared using grease-sealed, vacuum-packed capillaries. Details on single-crystal X-ray crystallography studies are reported in the Supporting Information.

[Eu(thf)_3_]_2_(µ-η^1^:η^1^-N_2_Ph_2_)_2_ (**1)**: Yield: 0.48 g (44%); m.p.: 90–95 °C; ^1^H NMR (300 MHz): 7.05 m (8H), 6.76 m (4H), 6.62 m (4H), 3.57 s (24H), 1.72 s (24H); IR (Nujol): ν^−1^ (cm^−1^) = 2728 (s), 2664 (s), 1579 (s), 1456 (s), 1380 (s), 1293 (s), 1252 (s), 1153 (s), 1071 (s), 1024 (s), 978 (s).

[Eu(dme)_2_]_2_(µ-η^2^:η^2^N_2_Ph_2_)_2_ (**2**): Yield: 0.68 g (73%); m.p.: 120 °C (decomp.); ^1^H NMR (300 MHz): 7.05 m (8H), 6.76 m (8H), 6.62 m (4H), 3.43 t (16H), 3.27 (24H); IR (Nujol): ν^−1^ (cm^−1^) = 2714 (s), 1579 (s), 1451 (s), 1376 (s), 1304 (s), 1153 (s), 1071 (s), 968 (s), 725 (s).

Solvents were obtained from a vacuum atmosphere solvent system and were degassed twice before use. Azobenzene was acquired commercially and dried under vaccum. Compounds **1** and **2** were synthesized by solvothermal means. 2.0 mmols (0.30 g) of europium filings were combined with 2.0 mmols (3.6 g) of azobenzene with either 10 mL of THF or DME. Compound **1** was formed after four days of sonication, X-ray quality crystals were obtained by slow cooling of the reaction vessel (0.36 g, 70% yield). Compound **2** was sonicated for 48 h, X-ray quality crystals were obtained by slow cooling for the reaction vessel.

### 4.2. Single-Crystal X-Ray Diffraction

All crystals suitable for single-crystal X-ray diffraction were removed from a vial or a Schlenk under inert gas and immediately covered with a layer of silicone oil. A single crystal was selected, mounted on a glass rod, and placed in the cold N_2_ stream by an Oxford Cryosystems low-temperature device (−186 °C). The crystallographic data collection was performed for compounds **1** and **2** on a Bruker Kappa Duo diffractometer with Mo Kα radiation (λ = 0.71073 Å) and an Apex II CCD area detector [32]. The program CELL NOW determined the unit cell constants and the orientation matrices [33]. Data integration was carried out using SAINT [32]. Empirical absorption corrections were applied using SADABS [34,35]. The structures were solved with the use of the intrinsic phasing option in SHELXT and refined by the full-matrix least-squares procedures in SHELXL [36,37,38,39,40,41]. The space group assignments and structural solutions were evaluated using PLATON [42,43]. All non-hydrogen atoms were refined anisotropically. Hydrogen atoms were placed in calculated positions corresponding to standard bond lengths and angles and refined using a riding model. Centroids and planes were determined by features of the programs Mercury and Diamond [44,45]. All values for published compounds were based on a Cambridge Structural Database search, and all values for presented and published compounds fall within expected ranges [46,47].

### 4.3. Computational Methods

Density functional theory (DFT) calculations were performed with the B3LYP [48], CAM-B3LYP [49], LC-ωHPBE [50], and ωB97XD [51] hybrid density functionals, inclusion of the GD2 [52] (ωB97XD) and GD3(BJ) [53] dispersion corrections (other three; GD3 not reported here due to the great similarity between its predictions with GD3(BJ)), and the Def2-SVP and Def2-TZV [54] basis sets for C/H/N/O atoms in conjunction with the MWB53 [55] effective core potential for Eu. Natural population analysis (NPA) [56] was performed for each theory level with the Def2-SVP and Def2-TZVP basis sets for Eu as obtained from the Basis Set Exchange [57]. All theory levels produced Ci-symmetric minima as characterized by normal mode analysis. Calculations were performed with the Gaussian09 ver. D.01 program [58] with program-option “ultrafine” grid sizes (integration grid of 99 radial shells and 590 angular points per shell) and “tight” convergence criteria (force criterion RMS < 1.0 × 10^−5^, density matrix RMS < 1.0 × 10^−8^). All geometries are provided as .xyz files in the Supplementary Materials (Table S2).

## 5. Conclusions

We have prepared two novel europium complexes using the aprotic ligand azobenzene and two different co-ligands. Crystallographic evidence confirms the reduction in bond order of azobenzene, with the resulting final structures determined by the steric bulk of neutral co-ligands and the availability of the europium coordination sphere to accommodate secondary non-covalent interactions. We are encouraged by the synthetic method, direct metalation, which offers a facile route to the target compounds. This synthetic strategy has the potential to be further expanded to one of our main focuses, the alkaline earth metals. Including azobenzene can further enhance the possible application of Eu-based materials, as azobenzene can serve as a photoswitch under ultraviolet (UV) light and change its isomerization [23]. Ongoing extensive work is focusing on UV-visible-light spectroscopic studies to provide further insight into the potential use of compounds **1** and **2** in biomedical applications.

## Figures and Tables

**Table 1:** A comparison of select properties, including reactivity of alkaline earth metals and divalent rare earth metals [7,8,9].

Metal Cation (2+)	Ionic Radii (Å)	Electronegativity (Pauling)	E° (V) (M^2+^ _(aq)_ + 2e^−^ = M_(s)_)
Ca	1.12	1.00	−2.87
Yb	1.14	1.10	−2.22
Sr	1.25	0.95	−2.89
Sm	1.26	1.17	−2.30
Eu	1.24	1.20	−1.99
Ba	1.42	0.89	−2.90

**Table 2:** Selected bond lengths and angles for compounds **1** and **2**.

Compound	N-N (Å) Ligand	Eu-N (Å) Ligand		Eu-O (Å) Donor	N-Eu-N (°) Ligand	Dihedral (ɸ), Ph-NN-Ph (°)
**[Eu(thf)_3_]_2_(N_2_Ph_2_)_2_**	1.471(3)	2.469(3) 2.675(2) 2.456(2) 2.675(2)	―	2.592(2)– 2.659(2)	33.00(7) 32.91(7)	83.52(1)
**[Eu(dme)_2_]_2_(N_2_Ph_2_)_2_**	1.472(8), 1.479(7)	Eu1 2.707(6) 2.494(6) 2.644(6) 2.499(6)	Eu2 2.455(6) 3.237(5) 2.445(6) 2.731(5)	2.620(5)– 2.733(5)	32.5(2) 33.2(2) 32.6(2)	75.5(7) 78.8(7)

**Table 3:** Selected non-covalent interactions for compounds **1** and **2**.

Compound	Eu···C(π) (Å)		Eu···C(π) (Å)	Eu···H-C (Å)	
**[Eu(thf)_3_]_2_(N_2_Ph_2_)_2_**	Eu1 3.19 (C1) 3.28 (C7)	Eu1 3.19 (C1) 3.28 (C7)	2.98–3.29	―	
**[Eu(dme)_2_]_2_(N_2_Ph_2_)_2_**	Eu1 3.09 (C1)	Eu2 3.13 (C19)	―	Eu1 ―	Eu2 3.21 (H34B, DME) 3.25 (H6, Ph_2_N_2_)

## Data Availability

The spectroscopic data and tabulated crystallographic data presented in this study are included in the Supplementary Material, which can be accessed online. Deposition numbers 2388605 and 2388606 contain the supplementary crystallographic data for this article. The data are provided free of charge by the joint Cambridge Crystallographic Data Centre and Fachin-formationszentrum Karlsruhe Access Structures service (www.ccdc.cam.ac.uk/structures) (accessed on 3 October 2024).

## Supplementary Materials

The following supporting information can be downloaded at: https://www.mdpi.com/article/10.3390/molecules29215187/s1, Figures S1 and S2: ^1^H-NMR of **1** and **2**, respectively; Table S1: Crystallographic details; Table S2: .xyz files of optimized geometries for **1**; Table S3. Bond length/interatomic distance differences with experiment for of all theoretical methods; Table S4. RMSDs, NPA charges, N=N vibrational mode energies, and method SCF timing reports for all levels of theory for **1**.
