# Supplementary material for: Azobenzene as an Effective Ligand in Europium Chemistry—A Synthetic and Theoretical Study
Source: Molecules. 2024 Nov 2;29(21):5187. doi: 10.3390/molecules29215187 (PMC11547820; doi:10.3390/molecules29215187)

checkCIF/PLATON report

Structure factors have been supplied for datablock(s) agg038x_a

THIS REPORT IS FOR GUIDANCE ONLY. IF USED AS PART OF A REVIEW PROCEDURE FOR PUBLICATION, IT SHOULD NOT REPLACE THE EXPERTISE OF AN EXPERIENCED CRYSTALLOGRAPHIC REFEREE.

No syntax errors found. CIF dictionary Interpreting this report

Datablock: agg038x_a

```
Bond precision:   C-C = 0.0107 A           Wavelength=0.71073

Cell:            a=10.4832(16)      b=10.8003(19)      c=19.632(3)
                 alpha=74.286(4)    beta=89.440(4)     gamma=77.989(4)
Temperature:     100 K

                  Calculated                   Reported
Volume            2090.3(6)                    2090.3(6)
Space group       P -1                         P -1
Hall group        -P 1                         -P 1
Moiety formula    C40 H60 Eu2 N4 O8            C40 H60 Eu2 N4 O8
Sum formula       C40 H60 Eu2 N4 O8            C40 H60 Eu2 N4 O8
Mr                1028.86                      1028.84
Dx,g cm-3         1.635                        1.635
Z                 2                            2
Mu (mm-1)         3.027                        3.027
F000              1036.0                       1036.0
F000'             1035.73
h,k,lmax          15,15,28                     15,15,28
Nref              12913                        12867
Tmin,Tmax         0.419,0.514                  0.603,0.746
Tmin'             0.365

Correction method= # Reported T Limits: Tmin=0.603 Tmax=0.746
AbsCorr = MULTI-SCAN

Data completeness= 0.996           Theta(max)= 30.639

                                                     wR2(reflections)=
R(reflections)= 0.0629( 6794)                        0.1088( 12867)

S = 0.957                 Npar= 495
```

The following ALERTS were generated. Each ALERT has the format
test-name_ALERT_alert-type_alert-level.
Click on the hyperlinks for more details of the test.

Alert level C

```
RINTA01_ALERT_3_C  The value of Rint is greater than 0.12
            Rint given   0.132
PLAT213_ALERT_2_C Atom C1              has ADP max/min Ratio .....        3.1 oblate
PLAT342_ALERT_3_C Low Bond Precision on  C-C Bonds ..............    0.01071 Ang.
PLAT906_ALERT_3_C Large K Value in the Analysis of Variance ......      4.704 Check
PLAT911_ALERT_3_C Missing FCF Refl Between Thmin & STh/L=    0.600          2 Report
               -2  2  0,   0  1  2,
PLAT977_ALERT_2_C Check Negative Difference Density on H18       .      -0.51 eA-3
PLAT977_ALERT_2_C Check Negative Difference Density on H20       .      -0.46 eA-3
PLAT977_ALERT_2_C Check Negative Difference Density on H22       .      -0.34 eA-3
PLAT977_ALERT_2_C Check Negative Difference Density on H24       .      -0.52 eA-3
PLAT977_ALERT_2_C Check Negative Difference Density on H25A      .      -0.49 eA-3
PLAT977_ALERT_2_C Check Negative Difference Density on H32C      .      -0.32 eA-3
PLAT977_ALERT_2_C Check Negative Difference Density on H40A      .      -0.37 eA-3
```

Alert level G

```
PLAT020_ALERT_3_G The Value of Rint is Greater Than 0.12 .........      0.132 Report
PLAT154_ALERT_1_G The s.u.'s on the Cell Angles are Equal ..(Note)      0.004 Degree
PLAT794_ALERT_5_G Tentative Bond Valency for Eu1       (II)      .       3.00 Info
PLAT794_ALERT_5_G Tentative Bond Valency for Eu2       (II)      .       2.90 Info
PLAT912_ALERT_4_G Missing # of FCF Reflections Above STh/L=  0.600         43 Note
PLAT933_ALERT_2_G Number of HKL-OMIT Records in Embedded .res File          1 Note
                0  1  2,
PLAT941_ALERT_3_G Average HKL Measurement Multiplicity ..........         2.4 Low
PLAT969_ALERT_5_G The 'Henn et al.' R-Factor-gap value ..........       1.00 Note
              Predicted wR2: Based on SigI**2 10.88 or SHELX Weight 11.60
PLAT978_ALERT_2_G Number C-C Bonds with Positive Residual Density.          0 Info
```

0 **ALERT level A** = Most likely a serious problem - resolve or explain
0 **ALERT level B** = A potentially serious problem, consider carefully
12 **ALERT level C** = Check. Ensure it is not caused by an omission or oversight
9 **ALERT level G** = General information/check it is not something unexpected

1 ALERT type 1 CIF construction/syntax error, inconsistent or missing data
10 ALERT type 2 Indicator that the structure model may be wrong or deficient
6 ALERT type 3 Indicator that the structure quality may be low
1 ALERT type 4 Improvement, methodology, query or suggestion
3 ALERT type 5 Informative message, check

It is advisable to attempt to resolve as many as possible of the alerts in all categories. Often the minor alerts point to easily fixed oversights, errors and omissions in your CIF or refinement strategy, so attention to these fine details can be worthwhile. In order to resolve some of the more serious problems it may be necessary to carry out additional measurements or structure refinements. However, the purpose of your study may justify the reported deviations and the more serious of these should normally be commented upon in the discussion or experimental section of a paper or in the "special_details" fields of the CIF. checkCIF was carefully designed to identify outliers and unusual parameters, but every test has its limitations and alerts that are not important in a particular case may appear. Conversely, the absence of alerts does not guarantee there are no aspects of the results needing attention. It is up to the individual to critically assess their own results and, if necessary, seek expert advice.

Publication of your CIF in IUCr journals

A basic structural check has been run on your CIF. These basic checks will be run on all CIFs submitted for publication in IUCr journals (*Acta Crystallographica*, *Journal of Applied Crystallography*, *Journal of Synchrotron Radiation*); however, if you intend to submit to *Acta Crystallographica Section C* or *E* or *IUCrData*, you should make sure that full publication checks are run on the final version of your CIF prior to submission.

Publication of your CIF in other journals

Please refer to the *Notes for Authors* of the relevant journal for any special instructions relating to CIF submission.

PLATON version of 06/01/2024; check.def file version of 05/01/2024

Datablock agg038x_a - ellipsoid plot

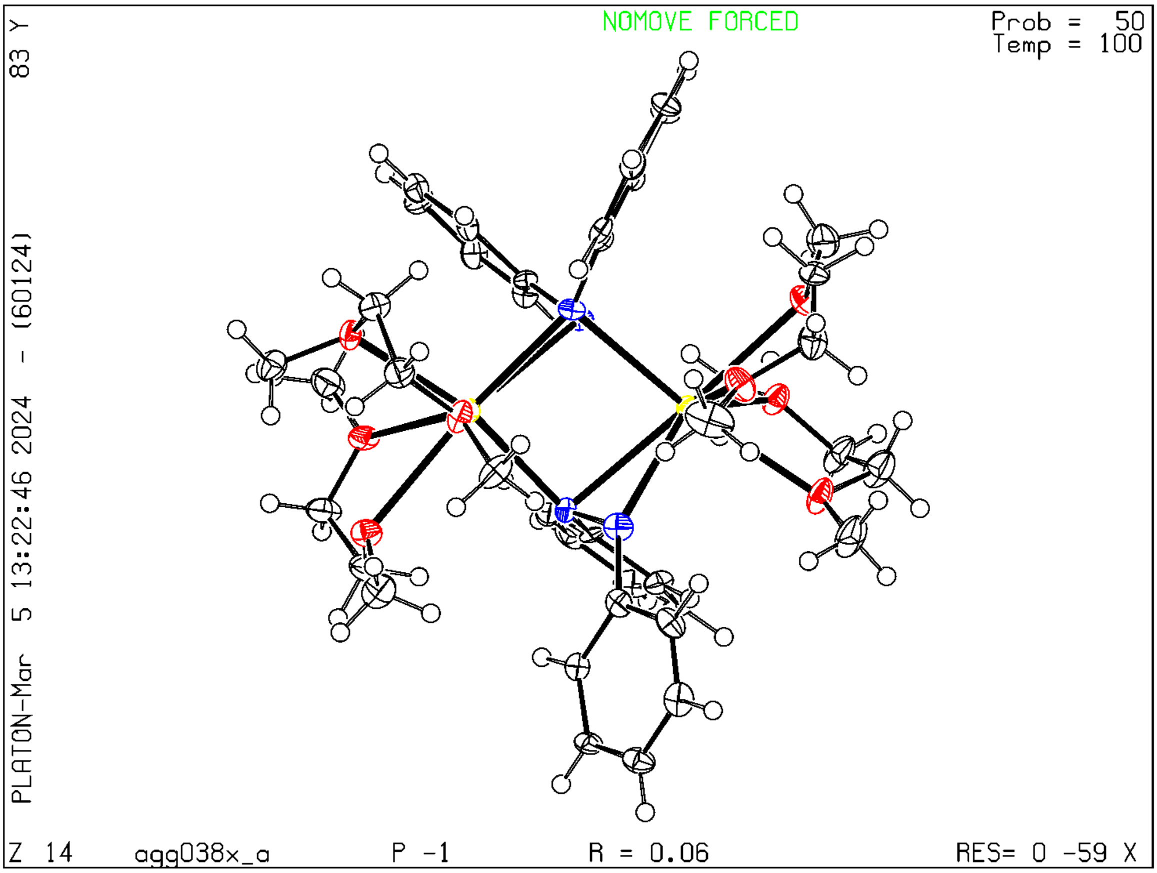

Supplement: Supplementary file 1 [file molecules-29-05187-s001.zip › EuAzoDME_CheckCif.pdf]
