# Supplementary material for: Azobenzene as an Effective Ligand in Europium Chemistry—A Synthetic and Theoretical Study
Source: Molecules. 2024 Nov 2;29(21):5187. doi: 10.3390/molecules29215187 (PMC11547820; doi:10.3390/molecules29215187)
Supplement: Supplementary file 1 [file molecules-29-05187-s001.zip › EuAzoSI.pdf]

Azobenzene as an effective ligand in Europium chemistry – a synthetic and theoretical study.

Damien G. Allis, Ana Torvisco, Cody C. Webb Jr., Miriam M. Gillett-Kunnath, Karin Ruhlandt-Senge

Content		Page
**Figure S1.**	^{1}H NMR spectrum of $[Eu(thf)_3]_2(Ph_2N_2)_2$	2
**Figure S2.**	^{1}H NMR spectrum of $[Eu(dme)_2]_2(Ph_2N_2)_2$	3
**Table S1.**	Crystallographic data and details of measurements for compounds **1** and **2**.	4
**Table S2.**	.xyz files of optimized geometries for structure **1**.	5-36
**Table S3.**	Bond length and interatomic distance differences with experiment for of all theoretical methods.	37
**Table S4.**	RMSDs, NPA charges, N=N vibrational mode energies, and method SCF timing reports for all levels of theory for **1**.	38

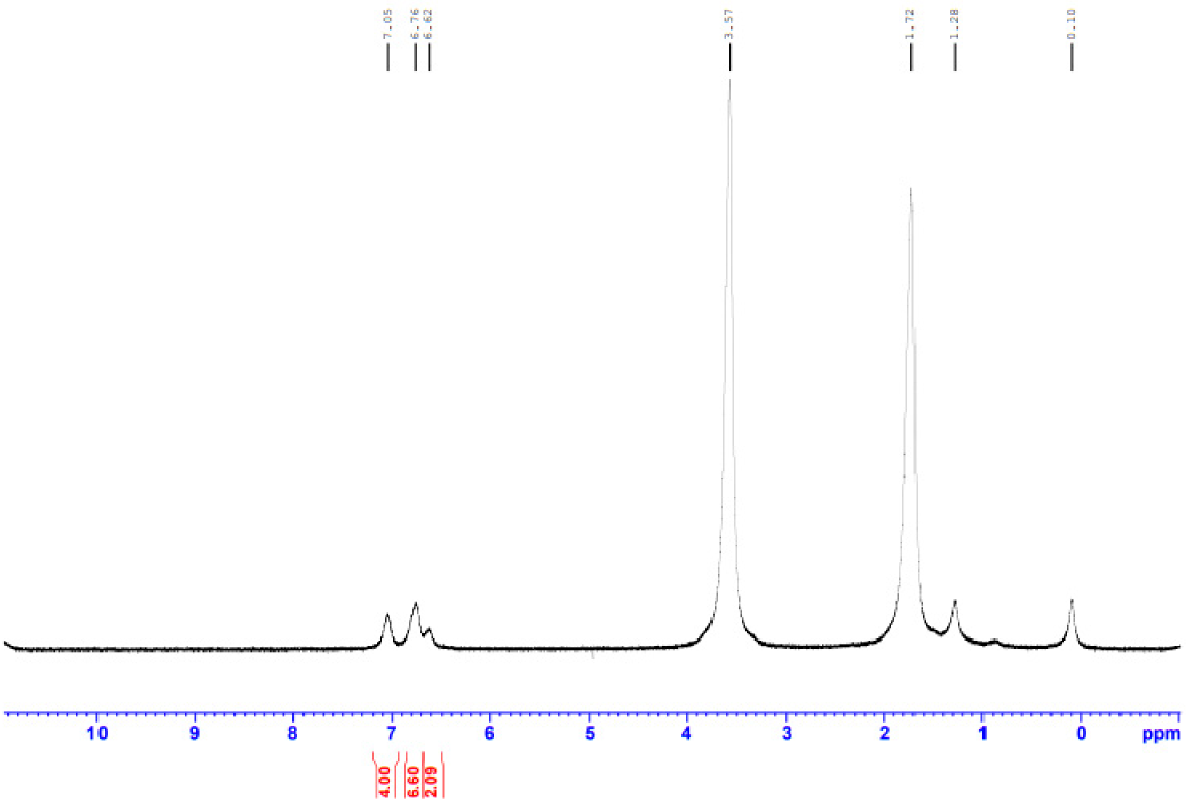

Figure S1. ^{1}H-NMR spectrum for $[Eu(thf)_3]_2(Ph_2N_2)_2$. Peaks from the phenyl substituents are integrated in the downfield region. Large peaks observed at 3.57 and 1.72 ppm are indicative of excess THF solvent and smaller peaks found at 1.28 and 0.10 ppm arose from vacuum grease contamination.

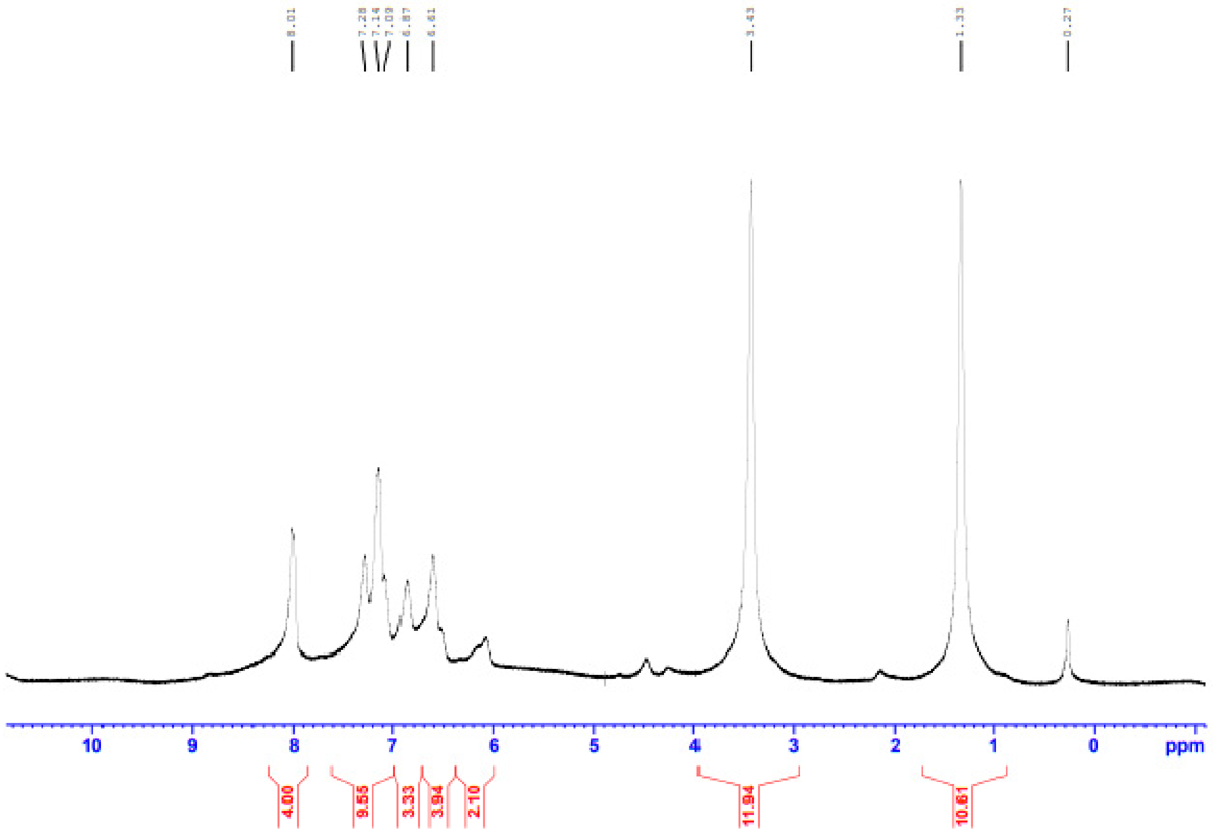

Figure S2. ^{1}H-NMR spectrum for $[Eu(dme)_2]_2(Ph_2N_2)_2$. Peaks from the phenyl substituents are integrated in the downfield region. Additional splitting is observed due to agostic interactions. Large peaks observed at 3.57 and 1.72 ppm are indicative of excess THF solvent and a smaller peak at 0.27 ppm arose from vacuum grease contamination.

Table S1. Crystallographic data and details of measurements for compounds **1** and **2**. Mo Kα (λ=0.71073Å). R1= Σ/ |F_o|- |F_c|/|Σ|F_d; wR2 = $[\Sigma_w(F_o^2-F_2^2)^2/\Sigma_w(F_o^2)^2]^{1/2}$

**Compound**	**[Eu(thf)$_3$]$_2$(N$_2$Ph$_2$)$_2$ AV11**	**[Eu(dme)$_2$]$_2$(N$_2$Ph$_2$)$_2$**
Formula	$C_{48}H_{68}N_4O_6Eu_2$	$C_{40}H_{60}N_4O_8Eu_2$
Fw (g mol^{-1})	1100.98	1028.84
a (Å)	10.9632(7)	10.4832(16)
b (Å)	13.5363(9)	10.8003(19)
c (Å)	15.7256(10)	19.632(3)
α (°)	90	74.286(4)
β (°)	91.565(1)	89.440(4)
γ (°)	90	77.989(4)
V (Å^3)	2332.8(3)	2090.3(6)
Z	2	2
Crystal size (mm)	0.36 × 0.3 × 0.26	0.32 × 0.25 × 0.22
Crystal habit	Block, orange	Block, orange
Crystal system	Monoclinic	Triclinic
Space group	*P*2$_1$/*n*	*P*-1
d_{calc} (Mg m^{-3})	1.567	1.635
μ (mm^{-1})	2.72	3.03
Radiation type	Mo K_α	Mo K_α
Wavelength (Å)	0.71073	0.71073
T (K)	100(2)	100(2)
θ range (°)	2.2–30.2	2.5–21.4
F(000)	1116	1036
T_{min}, T_{max}	0.601, 0.746	0.603, 0.746
R_{int}	0.021	0.132
No. of measured, independent and observed [$I > 2\sigma(I)$] reflections	16070, 4110, 3803	31350, 12867, 6794
independent reflections	4110	12867
No. of parameters, restraints	271, 0	495, 0
Δρmax, Δρmin (e Å^{-3})	1.57, -0.33	1.33, -1.35
R1, wR2 (all data)	R1 = 0.0266<br>wR2 = 0.0606	R1 = 0.1572<br>wR2 = 0.0865
R1, wR2 (>2*σ*)	R1 = 0.0240<br>wR2 = 0.0587	R1 = 0.0629<br>wR2 = 0.1088

Table S2. The following 32 pages contain the .xyz coordinates for the 16 optimized geometries of structure **1** (containing 128 atoms). Each .xyz file comment line contains the structure name and symmetry (Eu_thf_3_2_N2Ph2_2_AV11__Ci__), then (a) density functional, (b) dispersion correction, (c) C/H/O/N basis set, and (d) effective core potential for the Eu atom. For the first structure, the naming convention is as:
Eu_thf_3_2_N2Ph2_2_AV11__Ci__(a)b3lyp_(b)GD3BJ_(c)Def2SVP_(d)MWB53

```
128
Eu_thf_3_2_N2Ph2_2_AV11__Ci__b3lyp_GD3BJ_Def2SVP_MWB53
63       0.42935488      0.11494513     -1.74312512
8       -1.14896133      0.34916171     -3.82598076
8        2.13077209      1.51683188     -3.17047176
8        1.75830616     -1.54409971     -3.32838344
7       -0.68284006     -1.65411726      0.09905413
7        0.75234072     -1.58335311      0.07551566
6       -1.30392261     -2.46994817     -0.79186465
6       -0.64055539     -3.39378829     -1.65558984
1        0.43816450     -3.50923689     -1.55920063
6       -1.35971937     -4.16268322     -2.57308711
1       -0.81809173     -4.86883884     -3.21118083
6       -2.75079877     -4.06246142     -2.68582243
1       -3.30179413     -4.67263528     -3.40413972
6       -3.42239715     -3.16915338     -1.83174032
1       -4.51062831     -3.07306378     -1.89555536
6       -2.72842633     -2.39173052     -0.91473836
1       -3.25788683     -1.68541489     -0.26923921
6        1.44719278     -2.49742321      0.80490724
6        2.85153675     -2.29919035      0.99065641
1        3.30846779     -1.41807204      0.53105685
6        3.61563510     -3.18003445      1.74550780
1        4.68732725     -2.99004612      1.86172032
6        3.03416826     -4.29783108      2.36893843
1        3.63898352     -4.98592121      2.96292821
6        1.66230447     -4.51265980      2.19248307
1        1.18885359     -5.38603268      2.65244921
6        0.87730982     -3.64520314      1.43059042
1       -0.18153175     -3.84909033      1.28028297
6       -2.10081922     -0.70590629     -4.05658050
1       -1.90592129     -1.51447567     -3.34141679
1       -1.97595854     -1.09977373     -5.08271214
6       -3.44624471     -0.02316050     -3.87504320
1       -3.64605061      0.10101868     -2.79938663
1       -4.27606072     -0.59156041     -4.31854412
6       -3.20101275      1.33587722     -4.54695015
1       -3.83607205      2.13591681     -4.14579472
1       -3.38039211      1.26298118     -5.63154523
6       -1.71552148      1.61032375     -4.25933360
1       -1.16898754      1.95784342     -5.15200159
1       -1.57393405      2.33999470     -3.45114823
6        2.08504459      1.39263329     -4.60286250
1        1.17171259      0.83545208     -4.85687304
1        2.95249311      0.80560590     -4.94785073
6        2.08869451      2.83126416     -5.16109519
1        1.31903644      2.97750017     -5.93294298
1        3.06323144      3.06466883     -5.61736569
6        1.85581096      3.70300229     -3.91533675
1        2.29852543      4.70599391     -4.00064982
1        0.78108643      3.81811953     -3.70565884
6        2.49339973      2.86194693     -2.81883623
1        3.59543374      2.96012162     -2.81552614
1        2.10794831      3.04430631     -1.80811160
6        1.29565720     -2.36072930     -4.41500609
1        1.49296207     -1.83146773     -5.36509185
1        0.21260265     -2.51029752     -4.30702712
6        2.08933792     -3.66173616     -4.31353111
1        2.20969427     -4.16059579     -5.28606020
1        1.58102247     -4.35989809     -3.63063736
6        3.41165512     -3.18730870     -3.69859240
```

1	3.97318371	-3.98859707	-3.19744782
1	4.06036559	-2.74599767	-4.47311641
6	2.93516867	-2.11675703	-2.72102089
1	2.65884414	-2.54075463	-1.74270800
1	3.66529561	-1.30857852	-2.55893596
63	-0.40815175	0.14138299	1.80540637
8	1.17016446	-0.09283358	3.88826201
8	-2.10956897	-1.26050375	3.23275301
8	-1.73710303	1.80042783	3.39066469
7	0.70404319	1.91044539	-0.03677288
7	-0.73113759	1.83968124	-0.01323441
6	1.32512573	2.72627630	0.85414590
6	0.66175851	3.65011642	1.71787109
1	-0.41696137	3.76556501	1.62148188
6	1.38092249	4.41901135	2.63536836
1	0.83929486	5.12516696	3.27346208
6	2.77200189	4.31878954	2.74810368
1	3.32299726	4.92896341	3.46642097
6	3.44360027	3.42548150	1.89402157
1	4.53183143	3.32939190	1.95783661
6	2.74962945	2.64805865	0.97701961
1	3.27908996	1.94174301	0.33152046
6	-1.42598965	2.75375134	-0.74262599
6	-2.83033363	2.55551848	-0.92837516
1	-3.28726466	1.67440017	-0.46877560
6	-3.59443198	3.43636258	-1.68322655
1	-4.66612413	3.24637425	-1.79943907
6	-3.01296514	4.55415921	-2.30665718
1	-3.61778040	5.24224934	-2.90064696
6	-1.64110134	4.76898792	-2.13020182
1	-1.16765046	5.64236081	-2.59016796
6	-0.85610669	3.90153126	-1.36830917
1	0.20273487	4.10541846	-1.21800172
6	2.12202235	0.96223442	4.11886175
1	1.92712442	1.77080380	3.40369804
1	1.99716166	1.35610186	5.14499339
6	3.46744783	0.27948862	3.93732445
1	3.66725374	0.15530945	2.86166788
1	4.29726385	0.84788854	4.38082537
6	3.22221587	-1.07954910	4.60923140
1	3.85727518	-1.87958868	4.20807597
1	3.40159524	-1.00665306	5.69382648
6	1.73672460	-1.35399562	4.32161485
1	1.19019067	-1.70151529	5.21428284
1	1.59513718	-2.08366657	3.51342948
6	-2.06384147	-1.13630517	4.66514375
1	-1.15050946	-0.57912395	4.91915429
1	-2.93128999	-0.54927778	5.01013198
6	-2.06749138	-2.57493603	5.22337644
1	-1.29783332	-2.72117204	5.99522423
1	-3.04202832	-2.80834070	5.67964694
6	-1.83460784	-3.44667417	3.97761800
1	-2.27732231	-4.44966578	4.06293107
1	-0.75988330	-3.56179140	3.76794009
6	-2.47219661	-2.60561881	2.88111748
1	-3.57423061	-2.70379350	2.87780739
1	-2.08674519	-2.78797818	1.87039285
6	-1.27445408	2.61705742	4.47728734
1	-1.47175895	2.08779585	5.42737310
1	-0.19139952	2.76662564	4.36930837
6	-2.06813480	3.91806428	4.37581236
1	-2.18849114	4.41692392	5.34834145
1	-1.55981935	4.61622622	3.69291861
6	-3.39045199	3.44363682	3.76087365
1	-3.95198059	4.24492520	3.25972907
1	-4.03916246	3.00232579	4.53539766
6	-2.91396554	2.37308515	2.78330214
1	-2.63764102	2.79708276	1.80498925
1	-3.64409248	1.56490665	2.62121721

```
128
Eu_thf_3_2_N2Ph2_2_AV11__Ci__b3lyp_GD3BJ_Def2TZV_MWB53
63      0.47383481      0.11897488     -1.72781155
8      -1.13318351      0.33244894     -3.76700651
8       2.16833927      1.51431581     -3.12027874
8       1.75243381     -1.53072099     -3.32979671
7      -0.70610288     -1.67232549      0.09570453
7       0.77968284     -1.59588640      0.07312435
6      -1.31717513     -2.51967432     -0.79717574
6      -0.64470011     -3.44117091     -1.64809751
1       0.42657228     -3.53113543     -1.55928021
6      -1.35432335     -4.23083890     -2.54952116
1      -0.81363247     -4.93309210     -3.17404594
6      -2.74519155     -4.14920978     -2.65630754
1      -3.28585576     -4.77208667     -3.35471516
6      -3.42576778     -3.25538810     -1.81403539
1      -4.50473359     -3.17808287     -1.87454281
6      -2.73923728     -2.45957824     -0.91204183
1      -3.26713664     -1.75849990     -0.27800706
6       1.46983723     -2.53945757      0.79771345
6       2.87144354     -2.34796387      0.98290939
1       3.32222719     -1.46568339      0.54550582
6       3.63299632     -3.24453577      1.71524039
1       4.69556909     -3.06557297      1.83166183
6       3.04753524     -4.36968112      2.31595174
1       3.64555032     -5.06725840      2.88523441
6       1.67642842     -4.57604288      2.14100731
1       1.20725846     -5.44892413      2.58006554
6       0.89556544     -3.69123846      1.40126938
1      -0.15503307     -3.88173149      1.25248526
6      -2.12270878     -0.73480813     -4.04005072
1      -1.94986109     -1.53711165     -3.33208722
1      -1.97362081     -1.10047610     -5.05887516
6      -3.45966253     -0.02703343     -3.87520346
1      -3.68282707      0.07785494     -2.81375367
1      -4.27686959     -0.56587215     -4.35162502
6      -3.19686903      1.35337527     -4.51645536
1      -3.82549685      2.13171855     -4.09299805
1      -3.37575998      1.30924977     -5.59190024
6      -1.71005851      1.62846383     -4.22295674
1      -1.15781010      1.95008593     -5.10567691
1      -1.56739515      2.34291368     -3.41914043
6       2.12398344      1.39290910     -4.59470865
1       1.22925551      0.82426207     -4.83338804
1       2.99912880      0.83266440     -4.92100793
6       2.09740411      2.83757117     -5.14256059
1       1.31263232      2.97428150     -5.88510936
1       3.04950864      3.08519996     -5.61303891
6       1.87110651      3.71983593     -3.89151008
1       2.30785110      4.71182879     -3.99687749
1       0.80775463      3.83051099     -3.68119467
6       2.52254438      2.90715304     -2.77840281
1       3.61079463      3.00519797     -2.77762220
1       2.12847473      3.08365723     -1.78386220
6       1.27637686     -2.40503914     -4.41973973
1       1.47411605     -1.90125543     -5.36801472
1       0.20720367     -2.54274009     -4.28987018
6       2.07711427     -3.70118687     -4.27683713
1       2.20742310     -4.21245420     -5.22947768
1       1.57259002     -4.37876356     -3.58869050
6       3.41158949     -3.22341411     -3.66863956
1       3.94115598     -4.01978838     -3.14882373
1       4.06402499     -2.82607228     -4.44822194
6       2.97072324     -2.11404711     -2.71311340
1       2.69701728     -2.49634244     -1.73179637
1       3.69429577     -1.30872783     -2.60422876
63     -0.45263169      0.13735325      1.79009280
8       1.15438664     -0.07612081      3.82928776
8      -2.14713615     -1.25798769      3.18255999
8      -1.73123068      1.78704912      3.39207796
7       0.72730601      1.92865361     -0.03342328
```

7	-0.75847971	1.85221452	-0.01084310
6	1.33837825	2.77600245	0.85945699
6	0.66590323	3.69749904	1.71037876
1	-0.40536916	3.78746355	1.62156146
6	1.37552648	4.48716703	2.61180241
1	0.83483560	5.18942023	3.23632719
6	2.76639468	4.40553790	2.71858879
1	3.30705889	5.02841480	3.41699641
6	3.44697091	3.51171623	1.87631664
1	4.52593671	3.43441100	1.93682406
6	2.76044040	2.71590636	0.97432308
1	3.28833977	2.01482802	0.34028831
6	-1.44863411	2.79578570	-0.73543220
6	-2.85024041	2.60429199	-0.92062814
1	-3.30102406	1.72201151	-0.48322457
6	-3.61179319	3.50086390	-1.65295914
1	-4.67436596	3.32190110	-1.76938058
6	-3.02633211	4.62600925	-2.25367049
1	-3.62434719	5.32358652	-2.82295316
6	-1.65522529	4.83237100	-2.07872606
1	-1.18605533	5.70525226	-2.51778429
6	-0.87436231	3.94756658	-1.33898813
1	0.17623619	4.13805961	-1.19020401
6	2.14391191	0.99113626	4.10233197
1	1.97106421	1.79343978	3.39436847
1	1.99482394	1.35680422	5.12115641
6	3.48086565	0.28336155	3.93748471
1	3.70403020	0.17847319	2.87603492
1	4.29807272	0.82220028	4.41390627
6	3.21807216	-1.09704714	4.57873661
1	3.84669998	-1.87539043	4.15527930
1	3.39696311	-1.05292165	5.65418149
6	1.73126163	-1.37213571	4.28523799
1	1.17901322	-1.69375780	5.16795816
1	1.58859827	-2.08658555	3.48142168
6	-2.10278031	-1.13658098	4.65698990
1	-1.20805238	-0.56793395	4.89566929
1	-2.97792568	-0.57633627	4.98328918
6	-2.07620098	-2.58124305	5.20484184
1	-1.29142920	-2.71795337	5.94739061
1	-3.02830551	-2.82887184	5.67532016
6	-1.84990339	-3.46350780	3.95379133
1	-2.28664797	-4.45550067	4.05915874
1	-0.78655151	-3.57418286	3.74347592
6	-2.50134126	-2.65082491	2.84068406
1	-3.58959150	-2.74886984	2.83990345
1	-2.10727160	-2.82732911	1.84614345
6	-1.25517374	2.66136726	4.48202098
1	-1.45291293	2.15758355	5.43029597
1	-0.18600054	2.79906821	4.35215143
6	-2.05591114	3.95751499	4.33911838
1	-2.18621997	4.46878233	5.29175893
1	-1.55138690	4.63509168	3.65097175
6	-3.39038636	3.47974223	3.73092081
1	-3.91995286	4.27611651	3.21110498
1	-4.04282186	3.08240040	4.51050319
6	-2.94952011	2.37037523	2.77539465
1	-2.67581416	2.75267057	1.79407762
1	-3.67309265	1.56505596	2.66651001

```
128
Eu_thf_3_2_N2Ph2_2_AV11__Ci__b3lyp__Def2SVP_MWB53
63       0.38034858      0.09644543     -1.76043087
8       -1.35975438      0.26191350     -3.80524774
8        1.99153873      1.61236915     -3.32157870
8        1.79594030     -1.56051647     -3.42853190
7       -0.68314674     -1.65659392      0.10353346
7        0.75608593     -1.60520837      0.07402787
6       -1.32245396     -2.52637884     -0.73026844
6       -0.68202805     -3.51122433     -1.54596217
1        0.40019600     -3.62279433     -1.47714134
6       -1.42383887     -4.35742562     -2.37544247
1       -0.89413019     -5.11185042     -2.96768235
6       -2.81919264     -4.28011248     -2.44919158
1       -3.38798933     -4.95518979     -3.09254378
6       -3.46902000     -3.31933368     -1.65400842
1       -4.56036625     -3.23602284     -1.68705717
6       -2.75177028     -2.46376822     -0.82634144
1       -3.27393345     -1.71428079     -0.22427589
6        1.45575164     -2.53016296      0.79570742
6        2.87298854     -2.36858627      0.92691572
1        3.33762661     -1.50439971      0.44246663
6        3.64859989     -3.26416524      1.65462769
1        4.72923531     -3.09937122      1.72229206
6        3.07007221     -4.37000208      2.30265681
1        3.68432402     -5.07587415      2.86643270
6        1.68655992     -4.54831845      2.18753321
1        1.21176840     -5.41124714      2.66701190
6        0.88899005     -3.66110285      1.45961904
1       -0.18052705     -3.84582159      1.36301116
6       -2.29591907     -0.76453732     -4.18116941
1       -2.25480102     -1.56578455     -3.43101580
1       -2.01436891     -1.18149968     -5.16784366
6       -3.63306432     -0.03823359     -4.24641108
1       -4.02750067      0.09665696     -3.22702302
1       -4.38478966     -0.58204286     -4.83761386
6       -3.23471547      1.31312515     -4.86287166
1       -3.89222160      2.13607831     -4.54991738
1       -3.26318682      1.25666950     -5.96299057
6       -1.79142794      1.52642487     -4.36573536
1       -1.10872518      1.80803948     -5.18627691
1       -1.72342229      2.28670741     -3.57507360
6        2.18547741      1.50358389     -4.73327978
1        1.38032701      0.87528754     -5.13821082
1        3.15027557      1.00064100     -4.93312055
6        2.18879619      2.95228786     -5.27271415
1        1.25538299      3.18422936     -5.80622127
1        3.01645796      3.10423871     -5.98178372
6        2.33726601      3.82593332     -4.00031786
1        3.11597100      4.59659735     -4.09725811
1        1.38912825      4.33124743     -3.76448822
6        2.66553577      2.80805837     -2.90524782
1        3.75415715      2.61149316     -2.84927392
1        2.29741847      3.06515241     -1.90469104
6        1.30563849     -2.40321993     -4.48751645
1        1.34753337     -1.84097378     -5.43897065
1        0.25845773     -2.66464749     -4.27528480
6        2.23235520     -3.61934242     -4.51043614
1        2.29991402     -4.07999995     -5.50721335
1        1.87090814     -4.38487649     -3.80521174
6        3.55826327     -3.02513029     -4.01779046
1        4.24623731     -3.77539615     -3.60124305
1        4.07825739     -2.50625938     -4.84049289
6        3.08156365     -2.02557430     -2.96459129
1        2.95663602     -2.49764204     -1.97576910
1        3.74651757     -1.15389336     -2.85460105
63      -0.35914546      0.15988270      1.82271212
8        1.38095750     -0.00558537      3.86752899
8       -1.97033560     -1.35604103      3.38385995
8       -1.77473718      1.81684460      3.49081315
7        0.70434986      1.91292204     -0.04125221
```

7	-0.73488281	1.86153650	-0.01174662
6	1.34365709	2.78270696	0.79254969
6	0.70323117	3.76755246	1.60824342
1	-0.37899287	3.87912246	1.53942259
6	1.44504200	4.61375374	2.43772372
1	0.91533332	5.36817855	3.02996360
6	2.84039576	4.53644061	2.51147283
1	3.40919246	5.21151791	3.15482503
6	3.49022312	3.57566180	1.71628967
1	4.58156937	3.49235096	1.74933842
6	2.77297341	2.72009635	0.88862269
1	3.29513657	1.97060891	0.28655714
6	-1.43454851	2.78649109	-0.73342617
6	-2.85178541	2.62491439	-0.86463447
1	-3.31642349	1.76072784	-0.38018538
6	-3.62739677	3.52049336	-1.59234644
1	-4.70803219	3.35569934	-1.66001081
6	-3.04886909	4.62633020	-2.24037556
1	-3.66312090	5.33220228	-2.80415145
6	-1.66535679	4.80464657	-2.12525196
1	-1.19056528	5.66757526	-2.60473065
6	-0.86778692	3.91743097	-1.39733779
1	0.20173018	4.10214972	-1.30072991
6	2.31712220	1.02086545	4.24345066
1	2.27600414	1.82211267	3.49329705
1	2.03557203	1.43782780	5.23012491
6	3.65426744	0.29456171	4.30869233
1	4.04870380	0.15967116	3.28930427
1	4.40599279	0.83837098	4.89989511
6	3.25591860	-1.05679703	4.92515291
1	3.91342473	-1.87975018	4.61219863
1	3.28438994	-1.00034138	6.02527182
6	1.81263107	-1.27009674	4.42801661
1	1.12992830	-1.55171136	5.24855816
1	1.74462541	-2.03037929	3.63735485
6	-2.16427428	-1.24725576	4.79556103
1	-1.35912389	-0.61895941	5.20049207
1	-3.12907244	-0.74431288	4.99540180
6	-2.16759306	-2.69595974	5.33499540
1	-1.23417986	-2.92790123	5.86850252
1	-2.99525484	-2.84791059	6.04406497
6	-2.31606289	-3.56960519	4.06259911
1	-3.09476787	-4.34026922	4.15953936
1	-1.36792513	-4.07491931	3.82676947
6	-2.64433265	-2.55173024	2.96752907
1	-3.73295403	-2.35516504	2.91155517
1	-2.27621535	-2.80882429	1.96697229
6	-1.28443537	2.65954806	4.54979770
1	-1.32633025	2.09730191	5.50125190
1	-0.23725461	2.92097562	4.33756605
6	-2.21115207	3.87567055	4.57271739
1	-2.27871090	4.33632807	5.56949460
1	-1.84970501	4.64120461	3.86749299
6	-3.53706014	3.28145841	4.08007171
1	-4.22503419	4.03172428	3.66352430
1	-4.05705427	2.76258751	4.90277414
6	-3.06036052	2.28190243	3.02687254
1	-2.93543289	2.75397017	2.03805035
1	-3.72531445	1.41022148	2.91688230

```
128
Eu_thf_3_2_N2Ph2_2_AV11__Ci__b3lyp__Def2TZV_MWB53
63      0.43361929      0.12732566     -1.75368007
8      -1.34265960      0.28788728     -3.74651744
8       2.09236626      1.60052237     -3.24268427
8       1.76975736     -1.53195447     -3.45088067
7      -0.68074047     -1.69186687      0.08906172
7       0.80899861     -1.61406287      0.04154702
6      -1.31201974     -2.59785936     -0.74049545
6      -0.66311186     -3.57353619     -1.55203163
1       0.41228842     -3.65266721     -1.50255404
6      -1.39641348     -4.44337664     -2.35796860
1      -0.86770874     -5.18820667     -2.94327689
6      -2.79183573     -4.39108538     -2.41365475
1      -3.35064717     -5.07864800     -3.03358737
6      -3.45015247     -3.43531219     -1.62363474
1      -4.53236134     -3.37464589     -1.64549866
6      -2.73944107     -2.56011606     -0.81615283
1      -3.25887285     -1.81858019     -0.22158679
6       1.52884805     -2.55959804      0.74710514
6       2.94165839     -2.37913055      0.86273839
1       3.37960492     -1.50240740      0.40083962
6       3.73752581     -3.27851962      1.55684834
1       4.80611070     -3.10384941      1.61582373
6       3.18137965     -4.40477715      2.18395388
1       3.80628624     -5.10917042      2.71583931
6       1.80097910     -4.59974203      2.08542821
1       1.35012648     -5.47098797      2.54766717
6       0.98468105     -3.70713299      1.39213835
1      -0.07403909     -3.89717853      1.30821484
6      -2.29328466     -0.78078143     -4.13678747
1      -2.25190427     -1.55458650     -3.37666391
1      -1.98478814     -1.19079991     -5.10242169
6      -3.63627361     -0.06429542     -4.23116866
1      -4.05430231      0.06562735     -3.23292484
1      -4.35538692     -0.61145963     -4.83983101
6      -3.25521391      1.30161278     -4.84477515
1      -3.95322645      2.08876491     -4.56684637
1      -3.23456842      1.23266286     -5.93397323
6      -1.84623781      1.58462817     -4.28858667
1      -1.15184980      1.92213025     -5.05833400
1      -1.85426897      2.29390517     -3.46695315
6       2.22808475      1.47407986     -4.71285052
1       1.25984194      1.15521159     -5.09350754
1       2.96230492      0.69919578     -4.92783346
6       2.65450256      2.86623383     -5.22960021
1       3.71373166      2.86815873     -5.49125892
1       2.09214181      3.16184818     -6.11451943
6       2.40025211      3.81042316     -4.03065413
1       3.05219287      4.68309232     -4.04243314
1       1.36492706      4.15309100     -4.01896445
6       2.65807459      2.90511152     -2.83004363
1       3.72701211      2.78175905     -2.63690017
1       2.15123962      3.18510639     -1.91355966
6       1.25411297     -2.42711211     -4.51319487
1       1.29884491     -1.88517121     -5.46019763
1       0.22118126     -2.66642212     -4.27439901
6       2.17648997     -3.65140056     -4.50591303
1       2.25997183     -4.10878563     -5.49124251
1       1.80014301     -4.40273565     -3.81100714
6       3.51747218     -3.08071295     -3.99835572
1       4.15857457     -3.84641783     -3.56386135
1       4.06130629     -2.60047120     -4.81442952
6       3.07402808     -2.04789675     -2.95941277
1       2.91385201     -2.49239767     -1.97816420
1       3.74634726     -1.19649852     -2.86865328
63     -0.41241616      0.12900247      1.81596132
8       1.36386273     -0.03155916      3.80879869
8      -2.07116313     -1.34419424      3.30496552
8      -1.74855424      1.78828259      3.51316192
7       0.70194359      1.94819500     -0.02678047
```

7	-0.78779549	1.87039099	0.02073423
6	1.33322286	2.85418748	0.80277670
6	0.68431499	3.82986431	1.61431288
1	-0.39108529	3.90899534	1.56483529
6	1.41761661	4.69970477	2.42024985
1	0.88891186	5.44453479	3.00555814
6	2.81303886	4.64741350	2.47593600
1	3.37185030	5.33497612	3.09586862
6	3.47135560	3.69164032	1.68591599
1	4.55356447	3.63097402	1.70777991
6	2.76064419	2.81644419	0.87843408
1	3.28007597	2.07490831	0.28386804
6	-1.50764492	2.81592617	-0.68482389
6	-2.92045526	2.63545867	-0.80045714
1	-3.35840180	1.75873553	-0.33855837
6	-3.71632269	3.53484774	-1.49456709
1	-4.78490757	3.36017754	-1.55354248
6	-3.16017653	4.66110527	-2.12167263
1	-3.78508312	5.36549855	-2.65355806
6	-1.77977598	4.85607015	-2.02314696
1	-1.32892336	5.72731609	-2.48538592
6	-0.96347793	3.96346111	-1.32985710
1	0.09524221	4.15350666	-1.24593359
6	2.31448778	1.03710956	4.19906872
1	2.27310740	1.81091462	3.43894516
1	2.00599126	1.44712804	5.16470294
6	3.65747674	0.32062354	4.29344991
1	4.07550543	0.19070078	3.29520609
1	4.37659005	0.86778776	4.90211226
6	3.27641704	-1.04528466	4.90705640
1	3.97442957	-1.83243679	4.62912762
1	3.25577154	-0.97633473	5.99625448
6	1.86744094	-1.32830004	4.35086792
1	1.17305292	-1.66580213	5.12061525
1	1.87547210	-2.03757705	3.52923440
6	-2.20688163	-1.21775173	4.77513177
1	-1.23863882	-0.89888347	5.15578879
1	-2.94110179	-0.44286766	4.99011471
6	-2.63329944	-2.60990570	5.29188146
1	-3.69252854	-2.61183061	5.55354017
1	-2.07093869	-2.90552006	6.17680068
6	-2.37904899	-3.55409504	4.09293538
1	-3.03098974	-4.42676420	4.10471439
1	-1.34372394	-3.89676287	4.08124570
6	-2.63687147	-2.64878339	2.89232488
1	-3.70580898	-2.52543092	2.69918142
1	-2.13003649	-2.92877826	1.97584091
6	-1.23290985	2.68344023	4.57547612
1	-1.27764179	2.14149934	5.52247888
1	-0.19997814	2.92275025	4.33668026
6	-2.15528684	3.90772869	4.56819428
1	-2.23876870	4.36511376	5.55352376
1	-1.77893988	4.65906377	3.87328839
6	-3.49626905	3.33704108	4.06063697
1	-4.13737144	4.10274595	3.62614260
1	-4.04010316	2.85679932	4.87671077
6	-3.05282495	2.30422487	3.02169402
1	-2.89264889	2.74872579	2.04044545
1	-3.72514413	1.45282665	2.93093453

```
128
Eu_thf_3_2_N2Ph2_2_AV11__Ci__camb3lyp_GD3BJ_Def2SVP_MWB53
63       0.38195448      0.10772993     -1.73377825
8       -1.19496782      0.33499452     -3.81645617
8        2.05378369      1.51674227     -3.17731419
8        1.72002717     -1.51793747     -3.34269233
7       -0.66743955     -1.65149971      0.10030087
7        0.76357999     -1.58828504      0.07550617
6       -1.28776455     -2.47297144     -0.77953965
6       -0.62867453     -3.39643243     -1.63687998
1        0.45091693     -3.50768492     -1.54662081
6       -1.34800310     -4.17669064     -2.53834743
1       -0.80610828     -4.88551156     -3.17227227
6       -2.73456459     -4.08839872     -2.64155215
1       -3.28658194     -4.71022634     -3.34797855
6       -3.40226834     -3.19340224     -1.79475626
1       -4.49113680     -3.10625268     -1.84908557
6       -2.70879066     -2.40464578     -0.89483838
1       -3.23873399     -1.69751806     -0.25159569
6        1.44810052     -2.49991217      0.81027881
6        2.85099425     -2.31995056      0.98928637
1        3.31841676     -1.45107898      0.51837583
6        3.60379141     -3.19956966      1.74780270
1        4.67779648     -3.02230435      1.85716780
6        3.01373251     -4.30054016      2.38136496
1        3.61165656     -4.99136232      2.97796139
6        1.64434242     -4.49768845      2.21163890
1        1.16132479     -5.36059402      2.67986338
6        0.86954607     -3.62986331      1.44764587
1       -0.19191685     -3.82349369      1.30438836
6       -2.13313022     -0.71188944     -4.08457715
1       -1.96564447     -1.52663970     -3.36960759
1       -1.97581747     -1.09866545     -5.10815091
6       -3.47937761     -0.02966119     -3.94580290
1       -3.71604990      0.08818257     -2.87780579
1       -4.29387338     -0.59305427     -4.42067622
6       -3.20979150      1.32798632     -4.59923470
1       -3.84614595      2.12940357     -4.20474100
1       -3.36974592      1.26688322     -5.68632507
6       -1.73139510      1.58732476     -4.28254454
1       -1.16348742      1.91237320     -5.16962842
1       -1.59788487      2.33182869     -3.48711817
6        2.05229971      1.38682236     -4.60029781
1        1.13643612      0.84907156     -4.88215167
1        2.91565457      0.77834487     -4.91569297
6        2.11381861      2.81852277     -5.16316521
1        1.33889625      2.99854629     -5.92090745
1        3.08789497      3.00284894     -5.63941265
6        1.94337192      3.70479144     -3.92300834
1        2.46065971      4.67024557     -4.00759941
1        0.88045729      3.90093785     -3.71873819
6        2.50826210      2.82355461     -2.82392620
1        3.61372641      2.84204181     -2.81386502
1        2.13365260      3.03613970     -1.81588183
6        1.27823986     -2.32820215     -4.43252501
1        1.46526779     -1.78850468     -5.37786276
1        0.19876507     -2.50178605     -4.33016611
6        2.09715473     -3.60842579     -4.34232767
1        2.22210002     -4.09975965     -5.31690101
1        1.60803710     -4.31978906     -3.66025314
6        3.40671759     -3.10823666     -3.73490273
1        3.98973218     -3.89689389     -3.24068284
1        4.03991416     -2.64938225     -4.51050307
6        2.91276410     -2.05587032     -2.75294698
1        2.65988387     -2.48853228     -1.77272587
1        3.62428193     -1.23088560     -2.59708398
63      -0.36075184      0.14860840      1.79605383
8        1.21616857     -0.07865634      3.87873482
8       -2.03258911     -1.26040125      3.23959113
8       -1.69883805      1.77427960      3.40495748
7        0.68866070      1.90783052     -0.03801915
```

7	-0.74235820	1.84462100	-0.01322934
6	1.30899350	2.72928828	0.84182976
6	0.64991676	3.65275149	1.69917966
1	-0.42967114	3.76403343	1.60890909
6	1.36925173	4.43297016	2.60067493
1	0.82736712	5.14179225	3.23460644
6	2.75580944	4.34463652	2.70390077
1	3.30783278	4.96643269	3.41035120
6	3.42350089	3.44963983	1.85709632
1	4.51236612	3.36245768	1.91144295
6	2.73001580	2.66092288	0.95715065
1	3.25995017	1.95379091	0.31390518
6	-1.42687087	2.75625338	-0.74800114
6	-2.82977195	2.57632200	-0.92698767
1	-3.29720749	1.70746462	-0.45606328
6	-3.58256026	3.45596005	-1.68548983
1	-4.65657154	3.27872101	-1.79483403
6	-2.99248531	4.55691351	-2.31906811
1	-3.59040250	5.24774673	-2.91565751
6	-1.62308696	4.75402679	-2.14937024
1	-1.14005526	5.61691719	-2.61760863
6	-0.84829937	3.88618849	-1.38538323
1	0.21317282	4.07979072	-1.24214677
6	2.15435141	0.96821703	4.14682876
1	1.98687054	1.78295975	3.43184783
1	1.99705691	1.35501080	5.17039829
6	3.50058536	0.28596450	4.00804639
1	3.73724416	0.16810528	2.94004838
1	4.31509577	0.84935152	4.48290302
6	3.23098751	-1.07167092	4.66149794
1	3.86732447	-1.87310280	4.26700451
1	3.39095645	-1.01055722	5.74858504
6	1.75258323	-1.33099021	4.34483056
1	1.18468124	-1.65602068	5.23192369
1	1.61905149	-2.07549915	3.54941386
6	-2.03111375	-1.13046559	4.66257345
1	-1.11524909	-0.59271675	4.94442723
1	-2.89446743	-0.52197870	4.97795479
6	-2.09264735	-2.56215794	5.22545760
1	-1.31773383	-2.74217604	5.98321031
1	-3.06673049	-2.74647209	5.70169557
6	-1.92219372	-3.44844169	3.98531212
1	-2.43948635	-4.41389220	4.06991146
1	-0.85927837	-3.64459541	3.78105478
6	-2.48707005	-2.56721597	2.88621418
1	-3.59253382	-2.58569795	2.87614510
1	-2.11245492	-2.77981303	1.87817532
6	-1.25705783	2.58457967	4.49476727
1	-1.44405570	2.04489588	5.44011929
1	-0.17758983	2.75819486	4.39238938
6	-2.07601321	3.86477620	4.40455773
1	-2.20096124	4.35612414	5.37912450
1	-1.58692841	4.57614185	3.72246343
6	-3.38557029	3.36453650	3.79716034
1	-3.96861519	4.15316585	3.30293286
1	-4.01874202	2.90567845	4.57277971
6	-2.89159810	2.31216544	2.81521881
1	-2.63874789	2.74481369	1.83498444
1	-3.60309407	1.48715673	2.65938345

```
128
Eu_thf_3_2_N2Ph2_2_AV11__Ci__camb3lyp_GD3BJ_Def2TZV_MWB53
63      0.44944044      0.11690446     -1.71454528
8      -1.17249229      0.32979315     -3.73907573
8       2.11875679      1.51082910     -3.12160933
8       1.71705802     -1.51410436     -3.33703661
7      -0.69210074     -1.67376937      0.08959497
7       0.78398061     -1.60354084      0.07291281
6      -1.30151689     -2.52983984     -0.78877466
6      -0.63117942     -3.45066097     -1.63047454
1       0.44076500     -3.53307995     -1.54756571
6      -1.33824385     -4.25282435     -2.51534354
1      -0.79612740     -4.95728635     -3.13462575
6      -2.72384882     -4.18447937     -2.61323738
1      -3.26378477     -4.81969959     -3.29961305
6      -3.40260379     -3.29003685     -1.78016719
1      -4.48179404     -3.22320473     -1.83235734
6      -2.71870669     -2.48212561     -0.89555070
1      -3.24730248     -1.77951414     -0.26520416
6       1.46692652     -2.54701042      0.79562422
6       2.86565994     -2.37084817      0.97317382
1       3.32326390     -1.49672069      0.52864312
6       3.61814470     -3.26662063      1.70537487
1       4.68211236     -3.09784354      1.81585232
6       3.02703915     -4.37873570      2.31111523
1       3.61982716     -5.07907154      2.88079425
6       1.65982486     -4.57124652      2.14230308
1       1.18382855     -5.43694554      2.58590906
6       0.88735423     -3.68547286      1.40414048
1      -0.16497261     -3.86796704      1.26143806
6      -2.13744720     -0.73648594     -4.04147450
1      -1.98521993     -1.53948855     -3.32987933
1      -1.95567666     -1.10104308     -5.05434358
6      -3.47963123     -0.03974109     -3.92131111
1      -3.73999828      0.06037640     -2.86889961
1      -4.27581336     -0.58164155     -4.42617504
6      -3.20383182      1.33803107     -4.54697686
1      -3.84397149      2.11329115     -4.13734084
1      -3.35661134      1.29953620     -5.62534887
6      -1.72976675      1.61261604     -4.21700626
1      -1.15695564      1.92840568     -5.08764799
1      -1.60851619      2.33592480     -3.41845211
6       2.09755517      1.38321357     -4.58413641
1       1.18366982      0.85501587     -4.83988193
1       2.95008051      0.78155373     -4.89287863
6       2.14973834      2.81933286     -5.13659749
1       1.38769997      2.98956669     -5.89389898
1       3.11992196      3.01862658     -5.58999339
6       1.94462976      3.71339411     -3.89860009
1       2.43263897      4.68041353     -3.99699512
1       0.88511146      3.87872781     -3.71009748
6       2.52979049      2.87497497     -2.77471790
1       3.62015406      2.92201316     -2.75185787
1       2.12730041      3.07446614     -1.78876950
6       1.25747353     -2.37773584     -4.42806030
1       1.44379549     -1.86346827     -5.37184832
1       0.19178477     -2.53748913     -4.30095406
6       2.07942253     -3.65558589     -4.29976738
1       2.21302119     -4.15751653     -5.25540746
1       1.59122538     -4.34625883     -3.61417992
6       3.40164306     -3.15846595     -3.69771372
1       3.94861073     -3.94566037     -3.18470161
1       4.04253148     -2.74566027     -4.47722362
6       2.94589721     -2.06559885     -2.73808182
1       2.69218092     -2.45625393     -1.75542381
1       3.65370381     -1.24693165     -2.63340344
63     -0.42823828      0.13941789      1.77682037
8       1.19369882     -0.07348749      3.80134424
8      -2.09755791     -1.25450276      3.18388063
8      -1.69583932      1.77042007      3.39931994
7       0.71329563      1.93009752     -0.02732148
```

7	-0.76278444	1.85986688	-0.01063341
6	1.32270483	2.78616870	0.85105043
6	0.65235586	3.70697833	1.69275344
1	-0.41958932	3.78938638	1.60984035
6	1.35941045	4.50914320	2.57762883
1	0.81728447	5.21359576	3.19691516
6	2.74501525	4.44080906	2.67552896
1	3.28494243	5.07602882	3.36191093
6	3.42378078	3.54637600	1.84245562
1	4.50297177	3.47955483	1.89464902
6	2.73989531	2.73846641	0.95782973
1	3.26849878	2.03586227	0.32748110
6	-1.44573612	2.80333902	-0.73333554
6	-2.84447296	2.62717818	-0.91086697
1	-3.30206695	1.75303937	-0.46634892
6	-3.59697401	3.52297005	-1.64302791
1	-4.66094317	3.35419449	-1.75348925
6	-3.00588142	4.63510317	-2.24874833
1	-3.59868089	5.33545479	-2.81839552
6	-1.63866375	4.82761162	-2.07995638
1	-1.16267838	5.69332485	-2.52354560
6	-0.86617779	3.94181849	-1.34183286
1	0.18614970	4.12431491	-1.19913841
6	2.15864464	0.99279818	4.10374751
1	2.00639057	1.79581532	3.39217369
1	1.97688941	1.35733051	5.11662843
6	3.50083727	0.29607681	3.98353934
1	3.76118402	0.19598784	2.93112028
1	4.29702229	0.83797648	4.48839915
6	3.22507258	-1.08171441	4.60917634
1	3.86521542	-1.85695454	4.19950868
1	3.37787190	-1.04324382	5.68754604
6	1.75100379	-1.35631409	4.27922753
1	1.17821504	-1.67213905	5.14987158
1	1.62975178	-2.07960235	3.48065293
6	-2.07635540	-1.12688420	4.64640775
1	-1.16247115	-0.59868548	4.90215134
1	-2.92888073	-0.52522435	4.95514997
6	-2.12853649	-2.56300248	5.19887178
1	-1.36650215	-2.73323157	5.95617755
1	-3.09872222	-2.76229913	5.65226168
6	-1.92341775	-3.45706545	3.96087698
1	-2.41141983	-4.42408765	4.05927269
1	-0.86389765	-3.62239016	3.77237754
6	-2.50858334	-2.61865244	2.83699282
1	-3.59894570	-2.66569853	2.81413055
1	-2.10608915	-2.81814451	1.85104624
6	-1.23624785	2.63403451	4.49035397
1	-1.42258280	2.11976063	5.43413629
1	-0.17055621	2.79377068	4.36325521
6	-2.05817507	3.91189815	4.36206761
1	-2.19177045	4.41382383	5.31771045
1	-1.56996358	4.60256842	3.67648658
6	-3.38040082	3.41480330	3.76000475
1	-3.92735474	4.20201008	3.24699746
1	-4.02129959	3.00200043	4.53950809
6	-2.92466828	2.32193693	2.80036534
1	-2.67094103	2.71259827	1.81771209
1	-3.63248564	1.50328064	2.69567661

```
128
Eu_thf_3_2_N2Ph2_2_AV11__Ci__camb3lyp__Def2SVP_MWB53
63      0.35832216      0.10469878     -1.74591026
8      -1.33535444      0.29011064     -3.79089190
8       2.03055996      1.54290223     -3.23977460
8       1.71796877     -1.51994836     -3.41940567
7      -0.65948138     -1.65642418      0.09444038
7       0.77335473     -1.59788572      0.06636207
6      -1.28229910     -2.51009504     -0.75763857
6      -0.62641005     -3.45924143     -1.59053083
1       0.45587999     -3.55833313     -1.51429738
6      -1.34931304     -4.28638197     -2.44731968
1      -0.80663492     -5.01579886     -3.05723190
6      -2.73857747     -4.22287188     -2.53117926
1      -3.29351660     -4.88408058     -3.19890354
6      -3.40344885     -3.29876321     -1.71461724
1      -4.49452835     -3.22850447     -1.75298302
6      -2.70593269     -2.46311903     -0.85996306
1      -3.23954077     -1.73960295     -0.23811300
6       1.46146620     -2.51303471      0.79866318
6       2.87245250     -2.35727086      0.94123591
1       3.34480083     -1.50322147      0.44841807
6       3.63197266     -3.24312377      1.68662655
1       4.71183916     -3.08345186      1.76309048
6       3.04312874     -4.33151684      2.34299890
1       3.64660234     -5.03052471      2.92473683
6       1.66644219     -4.50497461      2.21326942
1       1.18222595     -5.35789520      2.69886552
6       0.88421080     -3.62816791      1.46567909
1      -0.18374656     -3.80972721      1.35563215
6      -2.22966978     -0.76320633     -4.16120536
1      -2.11187945     -1.59176303     -3.45095166
1      -1.97994750     -1.12287896     -5.17708185
6      -3.59655597     -0.10539726     -4.12712214
1      -3.93554075     -0.02796450     -3.08339010
1      -4.35286239     -0.66535098     -4.69422709
6      -3.29861568      1.27911819     -4.71143144
1      -3.96334023      2.05973660     -4.32004773
1      -3.40201859      1.26424761     -5.80688998
6      -1.83856016      1.53593013     -4.30768198
1      -1.22148018      1.84449865     -5.16827978
1      -1.74433280      2.29347321     -3.51870556
6       2.09720795      1.42526702     -4.66297332
1       1.12873199      1.03731228     -5.01132016
1       2.87278529      0.69210405     -4.93810713
6       2.41089451      2.83314709     -5.19948325
1       1.75647613      3.11425322     -6.03606144
1       3.44818193      2.88293660     -5.56272627
6       2.23142894      3.73382810     -3.97220084
1       2.85912557      4.63509262     -4.00191569
1       1.18252119      4.04807074     -3.86041772
6       2.59923725      2.79209840     -2.83994359
1       3.69588632      2.68727613     -2.74231722
1       2.17674209      3.04651286     -1.86090110
6       1.25815679     -2.33463530     -4.50067792
1       1.35970491     -1.76819670     -5.44373542
1       0.19711309     -2.57046387     -4.34101597
6       2.15005291     -3.56941283     -4.48876123
1       2.24527242     -4.03129071     -5.48117843
1       1.74119304     -4.32309522     -3.79875064
6       3.46315419     -3.00940426     -3.94436023
1       4.11713007     -3.77429313     -3.50405423
1       4.02484184     -2.49988449     -4.74338498
6       2.96900343     -2.00616611     -2.91069980
1       2.79729433     -2.47537755     -1.92881019
1       3.64541387     -1.14853578     -2.77401247
63     -0.33711904      0.15162935      1.80819151
8       1.35655756     -0.03378251      3.85317315
8      -2.00935684     -1.28657411      3.30205585
8      -1.69676564      1.77627648      3.48168692
7       0.68068450      1.91275230     -0.03215913
```

7	-0.75215160	1.85421385	-0.00408082
6	1.30350222	2.76642316	0.81991982
6	0.64761317	3.71556955	1.65281208
1	-0.43467687	3.81466125	1.57657863
6	1.37051616	4.54271009	2.50960093
1	0.82783804	5.27212698	3.11951315
6	2.75978059	4.47920000	2.59346051
1	3.31471973	5.14040871	3.26118479
6	3.42465198	3.55509133	1.77689849
1	4.51573148	3.48483260	1.81526427
6	2.72713581	2.71944716	0.92224431
1	3.26074390	1.99593108	0.30039425
6	-1.44026308	2.76936283	-0.73638193
6	-2.85124937	2.61359899	-0.87895466
1	-3.32359771	1.75954959	-0.38613682
6	-3.61076953	3.49945189	-1.62434530
1	-4.69063604	3.33977999	-1.70080923
6	-3.02192561	4.58784497	-2.28071765
1	-3.62539922	5.28685283	-2.86245558
6	-1.64523907	4.76130273	-2.15098817
1	-1.16102283	5.61422333	-2.63658427
6	-0.86300767	3.88449603	-1.40339784
1	0.20494969	4.06605534	-1.29335090
6	2.25087290	1.01953445	4.22348661
1	2.13308258	1.84809115	3.51323291
1	2.00115063	1.37920709	5.23936310
6	3.61775910	0.36172538	4.18940339
1	3.95674387	0.28429263	3.14567135
1	4.37406552	0.92167910	4.75650834
6	3.31981880	-1.02279006	4.77371269
1	3.98454335	-1.80340847	4.38232898
1	3.42322172	-1.00791948	5.86917123
6	1.85976329	-1.27960200	4.36996323
1	1.24268330	-1.58817053	5.23056103
1	1.76553592	-2.03714508	3.58098681
6	-2.07600482	-1.16893889	4.72525457
1	-1.10752887	-0.78098416	5.07360141
1	-2.85158216	-0.43577592	5.00038838
6	-2.38969138	-2.57681897	5.26176450
1	-1.73527300	-2.85792510	6.09834269
1	-3.42697881	-2.62660848	5.62500752
6	-2.21022581	-3.47749997	4.03448209
1	-2.83792245	-4.37876449	4.06419694
1	-1.16131807	-3.79174261	3.92269897
6	-2.57803413	-2.53577028	2.90222484
1	-3.67468320	-2.43094801	2.80459847
1	-2.15553896	-2.79018474	1.92318235
6	-1.23695366	2.59096343	4.56295917
1	-1.33850178	2.02452482	5.50601667
1	-0.17590996	2.82679199	4.40329722
6	-2.12884978	3.82574095	4.55104248
1	-2.22406929	4.28761884	5.54345968
1	-1.71998991	4.57942334	3.86103189
6	-3.44195106	3.26573238	4.00664148
1	-4.09592694	4.03062126	3.56633548
1	-4.00363872	2.75621261	4.80566623
6	-2.94780031	2.26249423	2.97298105
1	-2.77609121	2.73170568	1.99109144
1	-3.62421075	1.40486391	2.83629372

```
128
Eu_thf_3_2_N2Ph2_2_AV11__Ci__camb3lyp__Def2TZV_MWB53
63       0.42721251      0.12489533     -1.73139692
8       -1.29653259      0.29488709     -3.72407524
8        2.09560380      1.55114973     -3.17469377
8        1.71907553     -1.50721587     -3.41189807
7       -0.67693077     -1.68491853      0.07866958
7        0.80149729     -1.61412085      0.04979728
6       -1.29277236     -2.57566812     -0.76596627
6       -0.62917465     -3.52167607     -1.58656429
1        0.44554233     -3.59046970     -1.52502368
6       -1.34321360     -4.37254932     -2.41989458
1       -0.80274840     -5.09700430     -3.01770970
6       -2.73154708     -4.32972909     -2.49027142
1       -3.27684740     -5.00447042     -3.13375759
6       -3.40404643     -3.40555001     -1.68569146
1       -4.48526340     -3.35507096     -1.71675972
6       -2.71276011     -2.54954090     -0.85206072
1       -3.24185913     -1.82863969     -0.24293920
6        1.49656158     -2.55717190      0.76825573
6        2.90218363     -2.39355817      0.90784729
1        3.35759081     -1.52862634      0.44328869
6        3.66879336     -3.28998981      1.62584930
1        4.73701505     -3.12873950      1.70401664
6        3.08797444     -4.39552515      2.25339733
1        3.69189786     -5.09922802      2.80748810
6        1.71471148     -4.57570326      2.12631410
1        1.24441175     -5.43561695      2.58746285
6        0.92749737     -3.68637998      1.40678851
1       -0.13018275     -3.86431738      1.29919534
6       -2.22712998     -0.77857526     -4.10286124
1       -2.13584218     -1.57532248     -3.37266789
1       -1.95421023     -1.14961756     -5.09313600
6       -3.58209771     -0.09279571     -4.11358172
1       -3.94882062      0.00275558     -3.09291517
1       -4.31911487     -0.64038381     -4.69688739
6       -3.26052537      1.28896843     -4.70812232
1       -3.94617556      2.05739611     -4.36173302
1       -3.31103663      1.25183930     -5.79628562
6       -1.82642915      1.57645012     -4.23981714
1       -1.18161002      1.90964942     -5.05186849
1       -1.78797696      2.29301144     -3.42679664
6        2.17875449      1.42416217     -4.63617536
1        1.21842891      1.04582800     -4.97679753
1        2.94764062      0.69390956     -4.87911357
6        2.50108274      2.83137761     -5.16938712
1        3.54160380      2.88924975     -5.48742946
1        1.87780249      3.09620726     -6.02066435
6        2.26372549      3.75669545     -3.96153224
1        2.87019183      4.65879684     -4.00166773
1        1.21591908      4.04713516     -3.89451697
6        2.62119549      2.86532516     -2.78382674
1        3.70162533      2.78840872     -2.64628856
1        2.14863550      3.12032796     -1.84294055
6        1.23112552     -2.37932987     -4.48765496
1        1.33257846     -1.84301516     -5.43217643
1        0.18394943     -2.59209519     -4.29692360
6        2.11751404     -3.62039180     -4.43655022
1        2.22104330     -4.09255320     -5.41114506
1        1.70005362     -4.35052413     -3.74454043
6        3.44703518     -3.07606348     -3.89420646
1        4.05728225     -3.84845635     -3.43214378
1        4.02521546     -2.61591447     -4.69621798
6        2.99395118     -2.02731759     -2.88423068
1        2.80246147     -2.45497611     -1.90227791
1        3.67552932     -1.18545450     -2.78779407
63      -0.40600939      0.13143280      1.79367817
8        1.31773571     -0.03855897      3.78635649
8       -2.07440067     -1.29482161      3.23697502
8       -1.69787240      1.76354400      3.47417932
7        0.69813389      1.94124666     -0.01638833
```

7	-0.78029416	1.87044898	0.01248397
6	1.31397548	2.83199624	0.82824752
6	0.65037778	3.77800419	1.64884554
1	-0.42433920	3.84679782	1.58730493
6	1.36441673	4.62887745	2.48217583
1	0.82395153	5.35333242	3.07999095
6	2.75275020	4.58605721	2.55255267
1	3.29805052	5.26079854	3.19603884
6	3.42524955	3.66187814	1.74797271
1	4.50646652	3.61139909	1.77904097
6	2.73396324	2.80586903	0.91434197
1	3.26306226	2.08496782	0.30522045
6	-1.47535846	2.81350003	-0.70597448
6	-2.88098051	2.64988630	-0.84556604
1	-3.33638769	1.78495447	-0.38100744
6	-3.64759024	3.54631793	-1.56356805
1	-4.71581192	3.38506762	-1.64173539
6	-3.06677132	4.65185328	-2.19111608
1	-3.67069474	5.35555614	-2.74520685
6	-1.69350835	4.83203139	-2.06403285
1	-1.22320863	5.69194507	-2.52518160
6	-0.90629424	3.94270811	-1.34450726
1	0.15138588	4.12064550	-1.23691409
6	2.24833310	1.03490338	4.16514249
1	2.15704530	1.83165060	3.43494914
1	1.97541335	1.40594568	5.15541725
6	3.60330084	0.34912383	4.17586297
1	3.97002374	0.25357255	3.15519642
1	4.34031800	0.89671194	4.75916864
6	3.28172850	-1.03264030	4.77040357
1	3.96737869	-1.80106798	4.42401427
1	3.33223976	-0.99551118	5.85856687
6	1.84763228	-1.32012200	4.30209839
1	1.20281315	-1.65332130	5.11414974
1	1.80918008	-2.03668332	3.48907789
6	-2.15755137	-1.16783404	4.69845661
1	-1.19722579	-0.78949987	5.03907878
1	-2.92643750	-0.43758143	4.94139482
6	-2.47987962	-2.57504948	5.23166837
1	-3.52040067	-2.63292163	5.54971071
1	-1.85659936	-2.83987913	6.08294560
6	-2.24252237	-3.50036732	4.02381349
1	-2.84898871	-4.40246872	4.06394898
1	-1.19471596	-3.79080704	3.95679822
6	-2.59999237	-2.60899703	2.84610799
1	-3.68042221	-2.53208059	2.70856981
1	-2.12743238	-2.86399984	1.90522180
6	-1.20992239	2.63565799	4.54993621
1	-1.31137534	2.09934329	5.49445768
1	-0.16274630	2.84842332	4.35920485
6	-2.09631091	3.87671993	4.49883147
1	-2.19984017	4.34888133	5.47342631
1	-1.67885049	4.60685226	3.80682168
6	-3.42583206	3.33239161	3.95648771
1	-4.03607912	4.10478447	3.49442503
1	-4.00401234	2.87224259	4.75849923
6	-2.97274805	2.28364571	2.94651193
1	-2.78125834	2.71130423	1.96455916
1	-3.65432619	1.44178263	2.85007532

```
128
Eu_thf_3_2_N2Ph2_2_AV11__Ci__lcwpbe_GD3BJ_Def2SVP_MWB53
63       0.35003640      0.12767610     -1.74211998
8       -1.23112648      0.23811699     -3.84892280
8        2.04340948      1.58522613     -3.13316675
8        1.67815007     -1.52985262     -3.32560572
7       -0.70355533     -1.60104524      0.04340557
7        0.72084702     -1.58628294      0.04290524
6       -1.32011744     -2.46171508     -0.79778026
6       -0.65366529     -3.42805055     -1.59322250
1        0.42560495     -3.53462313     -1.47929763
6       -1.36160496     -4.24760551     -2.46422499
1       -0.81379233     -4.98905580     -3.05432407
6       -2.74359099     -4.15926595     -2.59335162
1       -3.28767374     -4.81335637     -3.27681807
6       -3.41795054     -3.22237790     -1.80413614
1       -4.50546850     -3.13224740     -1.88043352
6       -2.73534507     -2.39260697     -0.93709604
1       -3.27041520     -1.64643461     -0.34350985
6        1.34699848     -2.46854145      0.85556196
6        2.75342398     -2.35415639      1.04409838
1        3.27607941     -1.55285292      0.51463442
6        3.44329081     -3.21825166      1.87136350
1        4.52583266     -3.10006614      1.97802058
6        2.78398607     -4.22906829      2.57856208
1        3.33466707     -4.90850879      3.23141808
6        1.40891182     -4.35244313      2.41058400
1        0.87047970     -5.14542615      2.93896414
6        0.69539577     -3.50182538      1.57470646
1       -0.37474994     -3.64118141      1.42555678
6       -2.12771313     -0.84918676     -4.05762102
1       -1.88108011     -1.65369745     -3.35243993
1       -2.01658875     -1.23360800     -5.08748553
6       -3.48969350     -0.23138304     -3.83775936
1       -3.65893759     -0.11226881     -2.75784404
1       -4.30578359     -0.83937919     -4.24849803
6       -3.32666602      1.12224013     -4.51995970
1       -3.96654110      1.90158396     -4.08965438
1       -3.56332419      1.04210387     -5.59084886
6       -1.84528364      1.44449150     -4.31651523
1       -1.35828289      1.75903143     -5.25329608
1       -1.68632218      2.22511243     -3.55973473
6        1.88252640      1.72795391     -4.53095876
1        1.09369684      1.03773837     -4.85814689
1        2.82140592      1.45843803     -5.04914199
6        1.54837010      3.19871602     -4.70472227
1        0.48543934      3.36180111     -4.47972289
1        1.74499300      3.56424186     -5.72121673
6        2.42315661      3.86631134     -3.64079047
1        3.34947410      4.26371130     -4.07774748
1        1.90493609      4.70226362     -3.15445976
6        2.73274518      2.73486615     -2.65105322
1        3.81295164      2.51650080     -2.61188588
1        2.37618714      2.91988604     -1.63103318
6        1.25554737     -2.37130349     -4.39253336
1        1.42901362     -1.84860751     -5.34868932
1        0.18069190     -2.57007333     -4.28788159
6        2.09874420     -3.62940630     -4.27645519
1        2.24562209     -4.12939375     -5.24264453
1        1.61724827     -4.34290406     -3.59237027
6        3.38413853     -3.09682099     -3.66000087
1        3.97817433     -3.86670404     -3.15113992
1        4.01630533     -2.62771598     -4.42906377
6        2.84946386     -2.05363265     -2.69805886
1        2.56834689     -2.49066739     -1.72682410
1        3.54667808     -1.22219400     -2.51748509
63      -0.32883327      0.12865203      1.80440123
8        1.25232960      0.01821113      3.91120405
8       -2.02220635     -1.32889801      3.19544800
8       -1.65694695      1.78618075      3.38788697
7        0.72475846      1.85737337      0.01887568
```

7	-0.69964389	1.84261106	0.01937601
6	1.34132056	2.71804320	0.86006151
6	0.67486842	3.68437868	1.65550375
1	-0.40440183	3.79095126	1.54157888
6	1.38280809	4.50393363	2.52650624
1	0.83499546	5.24538392	3.11660532
6	2.76479412	4.41559407	2.65563287
1	3.30887687	5.06968450	3.33909932
6	3.43915367	3.47870603	1.86641739
1	4.52667163	3.38857552	1.94271477
6	2.75654819	2.64893509	0.99937729
1	3.29161832	1.90276274	0.40579110
6	-1.32579536	2.72486957	-0.79328071
6	-2.73222086	2.61048451	-0.98181713
1	-3.25487628	1.80918105	-0.45235317
6	-3.42208769	3.47457979	-1.80908225
1	-4.50462953	3.35639427	-1.91573933
6	-2.76278294	4.48539642	-2.51628083
1	-3.31346394	5.16483692	-3.16913683
6	-1.38770869	4.60877125	-2.34830275
1	-0.84927657	5.40175427	-2.87668289
6	-0.67419264	3.75815350	-1.51242521
1	0.39595306	3.89750953	-1.36327553
6	2.14891626	1.10551488	4.11990227
1	1.90228324	1.91002558	3.41472118
1	2.03779188	1.48993612	5.14976678
6	3.51089662	0.48771117	3.90004061
1	3.68014071	0.36859693	2.82012529
1	4.32698671	1.09570731	4.31077928
6	3.34786915	-0.86591200	4.58224095
1	3.98774422	-1.64525583	4.15193563
1	3.58452731	-0.78577574	5.65313011
6	1.86648676	-1.18816338	4.37879648
1	1.37948601	-1.50270331	5.31557733
1	1.70752531	-1.96878430	3.62201598
6	-1.86132327	-1.47162578	4.59324001
1	-1.07249371	-0.78141024	4.92042814
1	-2.80020279	-1.20210991	5.11142324
6	-1.52716698	-2.94238790	4.76700352
1	-0.46423622	-3.10547299	4.54200414
1	-1.72378987	-3.30791374	5.78349798
6	-2.40195348	-3.60998321	3.70307172
1	-3.32827098	-4.00738317	4.14002873
1	-1.88373296	-4.44593549	3.21674101
6	-2.71154205	-2.47853802	2.71333447
1	-3.79174852	-2.26017268	2.67416713
1	-2.35498402	-2.66355792	1.69331443
6	-1.23434424	2.62763161	4.45481461
1	-1.40781050	2.10493564	5.41097057
1	-0.15948878	2.82640145	4.35016284
6	-2.07754108	3.88573443	4.33873644
1	-2.22441896	4.38572188	5.30492578
1	-1.59604514	4.59923219	3.65465152
6	-3.36293540	3.35314912	3.72228212
1	-3.95697120	4.12303216	3.21342117
1	-3.99510220	2.88404411	4.49134502
6	-2.82826074	2.30996078	2.76034011
1	-2.54714377	2.74699551	1.78910535
1	-3.52547495	1.47852213	2.57976634

```
128
Eu_thf_3_2_N2Ph2_2_AV11__Ci__lcwpbe_GD3BJ_Def2TZV_MWB53
63      0.42737443      0.13085831     -1.71308234
8      -1.22033188      0.26627404     -3.73996295
8       2.18078936      1.52480738     -3.05986173
8       1.63341651     -1.53983727     -3.34470422
7      -0.70966358     -1.62335206      0.02727130
7       0.75456005     -1.60192322      0.04977596
6      -1.30900569     -2.52451602     -0.80845511
6      -0.62599272     -3.49812695     -1.56812793
1       0.44499303     -3.57954944     -1.45090223
6      -1.31523842     -4.34404882     -2.42178916
1      -0.76260524     -5.08926136     -2.98347681
6      -2.69407683     -4.27002452     -2.56249697
1      -3.22161215     -4.94152130     -3.22581217
6      -3.38474164     -3.32355495     -1.80625141
1      -4.46248780     -3.24886876     -1.89392295
6      -2.71759950     -2.47058143     -0.95555162
1      -3.25369104     -1.71950549     -0.38841867
6       1.37366481     -2.52602913      0.84469879
6       2.77602732     -2.42722318      1.02666406
1       3.29308294     -1.61950854      0.52282737
6       3.46037024     -3.31496779      1.82652509
1       4.53449338     -3.21299399      1.93265366
6       2.79340293     -4.33478358      2.50607783
1       3.33553286     -5.03082638      3.13125894
6       1.42007222     -4.44457574      2.34069201
1       0.88332047     -5.24077108      2.84461335
6       0.71487839     -3.56846043      1.53122993
1      -0.34780483     -3.68975076      1.38584132
6      -2.11182947     -0.85597092     -4.01408268
1      -1.87004343     -1.66061139     -3.32642838
1      -1.96066509     -1.19040837     -5.04323242
6      -3.48738708     -0.26060210     -3.81100244
1      -3.68764289     -0.18678916     -2.74287768
1      -4.27201627     -0.85719419     -4.27116540
6      -3.34525984      1.13047402     -4.43604226
1      -3.99303611      1.86389611     -3.96382650
1      -3.58536340      1.09515695     -5.49895873
6      -1.87103878      1.48688579     -4.22649022
1      -1.38459502      1.79020245     -5.15347350
1      -1.72960492      2.25578848     -3.47250528
6       1.95681127      1.68069483     -4.48597237
1       1.18745504      0.96986758     -4.77467601
1       2.87980898      1.45623203     -5.02628695
6       1.56237519      3.14005623     -4.62017426
1       0.52206139      3.26642195     -4.32384559
1       1.68655143      3.51881956     -5.63296863
6       2.48193706      3.83760022     -3.60989910
1       3.39970462      4.17257214     -4.09271569
1       2.00280906      4.70558608     -3.16250129
6       2.78501515      2.75989682     -2.56131290
1       3.85522547      2.59510682     -2.43686352
1       2.33455461      2.95063131     -1.59323189
6       1.19891368     -2.45846917     -4.38890160
1       1.34100904     -1.97010168     -5.35428578
1       0.14488779     -2.67152809     -4.23660519
6       2.08140265     -3.68730307     -4.23561335
1       2.22819903     -4.21009258     -5.17863854
1       1.63548416     -4.38006769     -3.52250108
6       3.37657448     -3.10991127     -3.66552845
1       3.97295523     -3.85480239     -3.14313794
1       3.98303783     -2.67430757     -4.46075841
6       2.86638048     -2.03134214     -2.72663597
1       2.61505770     -2.42756290     -1.74322245
1       3.53959685     -1.18334374     -2.61935490
63     -0.40617130      0.12546981      1.77536359
8       1.24153500     -0.00994591      3.80224420
8      -2.15958624     -1.26847925      3.12214298
8      -1.61221338      1.79616539      3.40698547
7       0.73086670      1.87968019      0.03500995
```

7	-0.73335692	1.85825135	0.01250529
6	1.33020882	2.78084414	0.87073636
6	0.64719584	3.75445508	1.63040918
1	-0.42378991	3.83587756	1.51318348
6	1.33644155	4.60037694	2.48407041
1	0.78380836	5.34558949	3.04575806
6	2.71527996	4.52635265	2.62477822
1	3.24281527	5.19784943	3.28809342
6	3.40594476	3.57988308	1.86853266
1	4.48369093	3.50519688	1.95620420
6	2.73880263	2.72690955	1.01783287
1	3.27489417	1.97583362	0.45069992
6	-1.35246169	2.78235726	-0.78241754
6	-2.75482419	2.68355131	-0.96438281
1	-3.27187982	1.87583666	-0.46054612
6	-3.43916711	3.57129592	-1.76424384
1	-4.51329025	3.46932212	-1.87037241
6	-2.77219980	4.59111170	-2.44379658
1	-3.31432974	5.28715451	-3.06897769
6	-1.39886909	4.70090387	-2.27841076
1	-0.86211734	5.49709921	-2.78233210
6	-0.69367527	3.82478855	-1.46894868
1	0.36900795	3.94607888	-1.32356007
6	2.13303260	1.11229904	4.07636393
1	1.89124655	1.91693951	3.38870963
1	1.98186821	1.44673650	5.10551367
6	3.50859020	0.51693022	3.87328369
1	3.70884602	0.44311729	2.80515893
1	4.29321940	1.11352231	4.33344665
6	3.36646296	-0.87414589	4.49832351
1	4.01423924	-1.60756799	4.02610775
1	3.60656653	-0.83882883	5.56123998
6	1.89224191	-1.23055767	4.28877147
1	1.40579815	-1.53387432	5.21575475
1	1.75080804	-1.99946035	3.53478653
6	-1.93560814	-1.42436671	4.54825362
1	-1.16625191	-0.71353945	4.83695726
1	-2.85860586	-1.19990391	5.08856820
6	-1.54117206	-2.88372810	4.68245551
1	-0.50085827	-3.01009382	4.38612684
1	-1.66534830	-3.26249144	5.69524988
6	-2.46073393	-3.58127210	3.67218035
1	-3.37850150	-3.91624402	4.15499694
1	-1.98160593	-4.44925795	3.22478254
6	-2.76381202	-2.50356870	2.62359415
1	-3.83402235	-2.33877869	2.49914477
1	-2.31335148	-2.69430319	1.65551314
6	-1.17771056	2.71479730	4.45118285
1	-1.31980592	2.22642981	5.41656703
1	-0.12368467	2.92785622	4.29888644
6	-2.06019953	3.94363119	4.29789460
1	-2.20699590	4.46642071	5.24091979
1	-1.61428103	4.63639582	3.58478233
6	-3.35537136	3.36623940	3.72780970
1	-3.95175210	4.11113051	3.20541919
1	-3.96183470	2.93063569	4.52303966
6	-2.84517736	2.28767027	2.78891722
1	-2.59385457	2.68389102	1.80550370
1	-3.51839372	1.43967187	2.68163615

128

Eu_thf_3_2_N2Ph2_2_AV11__Ci__lcwpbe__Def2SVP_MWB53

63	0.32143837	0.11529976	-1.74995739
8	-1.35106093	0.26243861	-3.82179839
8	1.91842656	1.62257592	-3.27671914
8	1.74080278	-1.52446211	-3.38733698
7	-0.68493545	-1.63024111	0.08005172
7	0.73955623	-1.58522543	0.05174383
6	-1.31050523	-2.48584197	-0.76291458
6	-0.65600034	-3.43206591	-1.59350201
1	0.42813832	-3.52717932	-1.51695834
6	-1.37906931	-4.26295169	-2.44325505
1	-0.83694008	-4.99333102	-3.05271565
6	-2.76577025	-4.20433822	-2.52233106
1	-3.32140893	-4.86960934	-3.18588879
6	-3.42863500	-3.28005777	-1.70798599
1	-4.52020102	-3.21210887	-1.74312242
6	-2.73184189	-2.44104368	-0.86089164
1	-3.26406834	-1.71462064	-0.24045343
6	1.41540272	-2.49423328	0.79861607
6	2.82194966	-2.34230047	0.95983867
1	3.30384221	-1.49155784	0.46961252
6	3.56637496	-3.22679146	1.71696958
1	4.64611628	-3.07133698	1.80616858
6	2.96542542	-4.30669625	2.37090516
1	3.55797260	-5.00605069	2.96393403
6	1.59215798	-4.47240516	2.22571660
1	1.09771671	-5.32024289	2.71035416
6	0.82502697	-3.59782439	1.46443377
1	-0.24332661	-3.77380017	1.33793728
6	-2.26323335	-0.77881148	-4.15764482
1	-2.13998776	-1.60326114	-3.44208312
1	-2.04444389	-1.15448831	-5.17416319
6	-3.61678185	-0.10401808	-4.09954155
1	-3.92698510	-0.00185029	-3.04948306
1	-4.39542249	-0.66249185	-4.63547904
6	-3.31137915	1.25798942	-4.71560688
1	-3.96555770	2.05524599	-4.34139707
1	-3.42254168	1.21663576	-5.80906201
6	-1.85088081	1.50587218	-4.32871599
1	-1.24250498	1.81660126	-5.19364901
1	-1.74778908	2.26482154	-3.54013417
6	2.10032157	1.52629151	-4.67723148
1	1.28016706	0.92243989	-5.08636854
1	3.04987191	1.00363364	-4.88913035
6	2.13191380	2.97047310	-5.19574641
1	1.20400727	3.22730240	-5.72349408
1	2.95919986	3.11383612	-5.90397914
6	2.30208344	3.81797787	-3.92567126
1	3.09393518	4.57298299	-4.01593760
1	1.36710727	4.33785028	-3.67769891
6	2.61197382	2.78644931	-2.85150243
1	3.69507879	2.56917982	-2.80240945
1	2.25577577	3.03930918	-1.84575966
6	1.30729274	-2.33899823	-4.47198659
1	1.43787709	-1.78022473	-5.41515762
1	0.24014847	-2.56803780	-4.34402673
6	2.18669837	-3.57773387	-4.43134056
1	2.31246524	-4.03820845	-5.42016763
1	1.75011872	-4.33015838	-3.75833477
6	3.47861690	-3.03005207	-3.84170630
1	4.11247434	-3.79964946	-3.38242659
1	4.07011076	-2.51813162	-4.61616405
6	2.95082472	-2.03447632	-2.82468320
1	2.72534135	-2.51318389	-1.85821474
1	3.63420840	-1.19167282	-2.64196458
63	-0.30023524	0.14102837	1.81223864
8	1.37226405	-0.00611048	3.88407964
8	-1.89722343	-1.36624780	3.33900039
8	-1.71959965	1.78079023	3.44961823
7	0.70613857	1.88656923	-0.01777047

7	-0.71835310	1.84155355	0.01053742
6	1.33170836	2.74217010	0.82519583
6	0.67720347	3.68839404	1.65578326
1	-0.40693520	3.78350745	1.57923959
6	1.40027243	4.51927981	2.50553630
1	0.85814320	5.24965915	3.11499690
6	2.78697338	4.46066634	2.58461231
1	3.34261205	5.12593746	3.24817004
6	3.44983813	3.53638589	1.77026724
1	4.54140414	3.46843700	1.80540367
6	2.75304502	2.69737181	0.92317289
1	3.28527147	1.97094876	0.30273468
6	-1.39419960	2.75056141	-0.73633482
6	-2.80074653	2.59862860	-0.89755742
1	-3.28263908	1.74788596	-0.40733127
6	-3.54517184	3.48311959	-1.65468833
1	-4.62491316	3.32766511	-1.74388733
6	-2.94422230	4.56302438	-2.30862391
1	-3.53676947	5.26237882	-2.90165278
6	-1.57095485	4.72873329	-2.16343535
1	-1.07651359	5.57657101	-2.64807291
6	-0.80382384	3.85415251	-1.40215252
1	0.26452974	4.03012830	-1.27565603
6	2.28443647	1.03513961	4.21992607
1	2.16119089	1.85958926	3.50436437
1	2.06564701	1.41081643	5.23644444
6	3.63798497	0.36034621	4.16182280
1	3.94818822	0.25817841	3.11176431
1	4.41662562	0.91881997	4.69776029
6	3.33258228	-1.00166129	4.77788813
1	3.98676082	-1.79891786	4.40367832
1	3.44374481	-0.96030763	5.87134326
6	1.87208393	-1.24954405	4.39099724
1	1.26370811	-1.56027314	5.25593026
1	1.76899220	-2.00849341	3.60241542
6	-2.07911844	-1.26996339	4.73951273
1	-1.25896394	-0.66611176	5.14864979
1	-3.02866879	-0.74730552	4.95141160
6	-2.11071067	-2.71414497	5.25802766
1	-1.18280415	-2.97097427	5.78577533
1	-2.93799673	-2.85750799	5.96626039
6	-2.28088031	-3.56164974	3.98795251
1	-3.07273205	-4.31665487	4.07821885
1	-1.34590414	-4.08152216	3.73998016
6	-2.59077069	-2.53012119	2.91378368
1	-3.67387567	-2.31285169	2.86469070
1	-2.23457264	-2.78298105	1.90804091
6	-1.28608961	2.59532636	4.53426784
1	-1.41667397	2.03655285	5.47743887
1	-0.21894535	2.82436593	4.40630798
6	-2.16549525	3.83406199	4.49362181
1	-2.29126211	4.29453658	5.48244888
1	-1.72891560	4.58648650	3.82061602
6	-3.45741378	3.28638020	3.90398755
1	-4.09127121	4.05597759	3.44470784
1	-4.04890763	2.77445975	4.67844530
6	-2.92962160	2.29080444	2.88696445
1	-2.70413822	2.76951202	1.92049599
1	-3.61300527	1.44800094	2.70424583

```
128
Eu_thf_3_2_N2Ph2_2_AV11__Ci__lcwpbe__Def2TZV_MWB53
63       0.35003640      0.12767610     -1.74211998
8       -1.23112648      0.23811699     -3.84892280
8        2.04340948      1.58522613     -3.13316675
8        1.67815007     -1.52985262     -3.32560572
7       -0.70355533     -1.60104524      0.04340557
7        0.72084702     -1.58628294      0.04290524
6       -1.32011744     -2.46171508     -0.79778026
6       -0.65366529     -3.42805055     -1.59322250
1        0.42560495     -3.53462313     -1.47929763
6       -1.36160496     -4.24760551     -2.46422499
1       -0.81379233     -4.98905580     -3.05432407
6       -2.74359099     -4.15926595     -2.59335162
1       -3.28767374     -4.81335637     -3.27681807
6       -3.41795054     -3.22237790     -1.80413614
1       -4.50546850     -3.13224740     -1.88043352
6       -2.73534507     -2.39260697     -0.93709604
1       -3.27041520     -1.64643461     -0.34350985
6        1.34699848     -2.46854145      0.85556196
6        2.75342398     -2.35415639      1.04409838
1        3.27607941     -1.55285292      0.51463442
6        3.44329081     -3.21825166      1.87136350
1        4.52583266     -3.10006614      1.97802058
6        2.78398607     -4.22906829      2.57856208
1        3.33466707     -4.90850879      3.23141808
6        1.40891182     -4.35244313      2.41058400
1        0.87047970     -5.14542615      2.93896414
6        0.69539577     -3.50182538      1.57470646
1       -0.37474994     -3.64118141      1.42555678
6       -2.12771313     -0.84918676     -4.05762102
1       -1.88108011     -1.65369745     -3.35243993
1       -2.01658875     -1.23360800     -5.08748553
6       -3.48969350     -0.23138304     -3.83775936
1       -3.65893759     -0.11226881     -2.75784404
1       -4.30578359     -0.83937919     -4.24849803
6       -3.32666602      1.12224013     -4.51995970
1       -3.96654110      1.90158396     -4.08965438
1       -3.56332419      1.04210387     -5.59084886
6       -1.84528364      1.44449150     -4.31651523
1       -1.35828289      1.75903143     -5.25329608
1       -1.68632218      2.22511243     -3.55973473
6        1.88252640      1.72795391     -4.53095876
1        1.09369684      1.03773837     -4.85814689
1        2.82140592      1.45843803     -5.04914199
6        1.54837010      3.19871602     -4.70472227
1        0.48543934      3.36180111     -4.47972289
1        1.74499300      3.56424186     -5.72121673
6        2.42315661      3.86631134     -3.64079047
1        3.34947410      4.26371130     -4.07774748
1        1.90493609      4.70226362     -3.15445976
6        2.73274518      2.73486615     -2.65105322
1        3.81295164      2.51650080     -2.61188588
1        2.37618714      2.91988604     -1.63103318
6        1.25554737     -2.37130349     -4.39253336
1        1.42901362     -1.84860751     -5.34868932
1        0.18069190     -2.57007333     -4.28788159
6        2.09874420     -3.62940630     -4.27645519
1        2.24562209     -4.12939375     -5.24264453
1        1.61724827     -4.34290406     -3.59237027
6        3.38413853     -3.09682099     -3.66000087
1        3.97817433     -3.86670404     -3.15113992
1        4.01630533     -2.62771598     -4.42906377
6        2.84946386     -2.05363265     -2.69805886
1        2.56834689     -2.49066739     -1.72682410
1        3.54667808     -1.22219400     -2.51748509
63      -0.32883327      0.12865203      1.80440123
8        1.25232960      0.01821113      3.91120405
8       -2.02220635     -1.32889801      3.19544800
8       -1.65694695      1.78618075      3.38788697
7        0.72475846      1.85737337      0.01887568
```

7	-0.69964389	1.84261106	0.01937601
6	1.34132056	2.71804320	0.86006151
6	0.67486842	3.68437868	1.65550375
1	-0.40440183	3.79095126	1.54157888
6	1.38280809	4.50393363	2.52650624
1	0.83499546	5.24538392	3.11660532
6	2.76479412	4.41559407	2.65563287
1	3.30887687	5.06968450	3.33909932
6	3.43915367	3.47870603	1.86641739
1	4.52667163	3.38857552	1.94271477
6	2.75654819	2.64893509	0.99937729
1	3.29161832	1.90276274	0.40579110
6	-1.32579536	2.72486957	-0.79328071
6	-2.73222086	2.61048451	-0.98181713
1	-3.25487628	1.80918105	-0.45235317
6	-3.42208769	3.47457979	-1.80908225
1	-4.50462953	3.35639427	-1.91573933
6	-2.76278294	4.48539642	-2.51628083
1	-3.31346394	5.16483692	-3.16913683
6	-1.38770869	4.60877125	-2.34830275
1	-0.84927657	5.40175427	-2.87668289
6	-0.67419264	3.75815350	-1.51242521
1	0.39595306	3.89750953	-1.36327553
6	2.14891626	1.10551488	4.11990227
1	1.90228324	1.91002558	3.41472118
1	2.03779188	1.48993612	5.14976678
6	3.51089662	0.48771117	3.90004061
1	3.68014071	0.36859693	2.82012529
1	4.32698671	1.09570731	4.31077928
6	3.34786915	-0.86591200	4.58224095
1	3.98774422	-1.64525583	4.15193563
1	3.58452731	-0.78577574	5.65313011
6	1.86648676	-1.18816338	4.37879648
1	1.37948601	-1.50270331	5.31557733
1	1.70752531	-1.96878430	3.62201598
6	-1.86132327	-1.47162578	4.59324001
1	-1.07249371	-0.78141024	4.92042814
1	-2.80020279	-1.20210991	5.11142324
6	-1.52716698	-2.94238790	4.76700352
1	-0.46423622	-3.10547299	4.54200414
1	-1.72378987	-3.30791374	5.78349798
6	-2.40195348	-3.60998321	3.70307172
1	-3.32827098	-4.00738317	4.14002873
1	-1.88373296	-4.44593549	3.21674101
6	-2.71154205	-2.47853802	2.71333447
1	-3.79174852	-2.26017268	2.67416713
1	-2.35498402	-2.66355792	1.69331443
6	-1.23434424	2.62763161	4.45481461
1	-1.40781050	2.10493564	5.41097057
1	-0.15948878	2.82640145	4.35016284
6	-2.07754108	3.88573443	4.33873644
1	-2.22441896	4.38572188	5.30492578
1	-1.59604514	4.59923219	3.65465152
6	-3.36293540	3.35314912	3.72228212
1	-3.95697120	4.12303216	3.21342117
1	-3.99510220	2.88404411	4.49134502
6	-2.82826074	2.30996078	2.76034011
1	-2.54714377	2.74699551	1.78910535
1	-3.52547495	1.47852213	2.57976634

```
128
Eu_thf_3_2_N2Ph2_2_AV11__Ci__wb97xd_Def2SVP_MWB53
63      0.36783064      0.10396828     -1.76834633
8      -1.12426263      0.33719707     -3.91842782
8       2.07539700      1.50896115     -3.18419699
8       1.73415023     -1.52565252     -3.33373956
7      -0.65547831     -1.63650119      0.11834229
7       0.77098347     -1.56381825      0.07466161
6      -1.28409906     -2.42287618     -0.78470375
6      -0.63728311     -3.33306653     -1.66862350
1       0.44255827     -3.46070304     -1.58152263
6      -1.37072530     -4.08243841     -2.58747390
1      -0.84073243     -4.78371266     -3.24037703
6      -2.75737909     -3.97037626     -2.68560626
1      -3.31975758     -4.56686998     -3.40603787
6      -3.41191792     -3.08293268     -1.81709960
1      -4.49934091     -2.97620718     -1.87042427
6      -2.70544040     -2.32545372     -0.89884400
1      -3.22185854     -1.62435087     -0.23679087
6       1.46801888     -2.45711228      0.81836950
6       2.86459296     -2.23690633      1.01245131
1       3.31125819     -1.35480104      0.54359320
6       3.63330830     -3.09751981      1.77920242
1       4.70097867     -2.89080319      1.90162282
6       3.06465562     -4.21492911      2.40787203
1       3.67482504     -4.88937957      3.01145026
6       1.70125360     -4.44970639      2.22449082
1       1.23673338     -5.32507450      2.68890197
6       0.91044326     -3.60158495      1.45165482
1      -0.14544577     -3.82280857      1.29545151
6      -2.09844584     -0.68554535     -4.13326825
1      -1.91947091     -1.49891311     -3.41683642
1      -1.99573202     -1.08988045     -5.15735774
6      -3.42202937      0.03031174     -3.94220402
1      -3.60831722      0.16886567     -2.86560588
1      -4.27011393     -0.51762793     -4.37440331
6      -3.14321348      1.37210997     -4.62391833
1      -3.75998547      2.19227853     -4.23595261
1      -3.31704343      1.29177578     -5.70833176
6      -1.65722963      1.60786970     -4.32758912
1      -1.09930454      1.96356694     -5.20917211
1      -1.50866518      2.32329821     -3.50507916
6       2.06811871      1.37885371     -4.60458301
1       1.19006827      0.77547478     -4.87767079
1       2.97113023      0.83575968     -4.92973731
6       2.01575498      2.81227876     -5.16856059
1       1.22038520      2.93457005     -5.91689987
1       2.96770686      3.06983754     -5.65649264
6       1.79510713      3.68159484     -3.92351934
1       2.23475394      4.68447928     -4.01217356
1       0.72259585      3.79482272     -3.70194683
6       2.44235796      2.84122742     -2.83582400
1       3.54401493      2.93986287     -2.84365247
1       2.06858771      3.02526322     -1.82031301
6       1.27463827     -2.33023738     -4.41549298
1       1.48437575     -1.80644542     -5.36557381
1       0.18831427     -2.46771749     -4.31784941
6       2.05038358     -3.63773308     -4.30672112
1       2.17148625     -4.13958358     -5.27641364
1       1.53027005     -4.32890479     -3.62583793
6       3.36905873     -3.17606692     -3.68549246
1       3.91634729     -3.97916968     -3.17413582
1       4.02935413     -2.74757819     -4.45627965
6       2.89546369     -2.09668493     -2.72120849
1       2.61258437     -2.51202919     -1.73985086
1       3.63040071     -1.29362026     -2.55967127
63     -0.34662752      0.15235984      1.83062758
8       1.14546576     -0.08086895      3.98070907
8      -2.05419388     -1.25263303      3.24647824
8      -1.71294711      1.78198065      3.39602081
7       0.67668143      1.89282932     -0.05606104
```

7	-0.74978035	1.82014637	-0.01238036
6	1.30530218	2.67920431	0.84698500
6	0.65848623	3.58939466	1.73090475
1	-0.42135514	3.71703116	1.64380388
6	1.39192843	4.33876653	2.64975515
1	0.86193555	5.04004078	3.30265828
6	2.77858221	4.22670439	2.74788751
1	3.34096070	4.82319811	3.46831912
6	3.43312105	3.33926080	1.87938085
1	4.52054403	3.23253530	1.93270552
6	2.72664352	2.58178185	0.96112525
1	3.24306167	1.88067900	0.29907212
6	-1.44681576	2.71344040	-0.75608825
6	-2.84338984	2.49323446	-0.95017006
1	-3.29005507	1.61112916	-0.48131195
6	-3.61210518	3.35384793	-1.71692117
1	-4.67977554	3.14713131	-1.83934157
6	-3.04345250	4.47125723	-2.34559078
1	-3.65362191	5.14570770	-2.94916901
6	-1.68005048	4.70603451	-2.16220957
1	-1.21553026	5.58140263	-2.62662072
6	-0.88924014	3.85791308	-1.38937357
1	0.16664890	4.07913670	-1.23317026
6	2.11964896	0.94187347	4.19554950
1	1.94067403	1.75524124	3.47911767
1	2.01693514	1.34620857	5.21963899
6	3.44323249	0.22601639	4.00448527
1	3.62952034	0.08746245	2.92788713
1	4.29131706	0.77395605	4.43668456
6	3.16441661	-1.11578184	4.68619958
1	3.78118860	-1.93595041	4.29823386
1	3.33824655	-1.03544765	5.77061301
6	1.67843275	-1.35154158	4.38987037
1	1.12050766	-1.70723882	5.27145336
1	1.52986831	-2.06697009	3.56736041
6	-2.04691559	-1.12252558	4.66686426
1	-1.16886514	-0.51914666	4.93995204
1	-2.94992711	-0.57943156	4.99201856
6	-1.99455186	-2.55595064	5.23084184
1	-1.19918208	-2.67824193	5.97918112
1	-2.94650373	-2.81350942	5.71877389
6	-1.77390400	-3.42526672	3.98580059
1	-2.21355081	-4.42815115	4.07445481
1	-0.70139273	-3.53849459	3.76422808
6	-2.42115484	-2.58489930	2.89810525
1	-3.52281181	-2.68353474	2.90593372
1	-2.04738458	-2.76893509	1.88259426
6	-1.25343515	2.58656550	4.47777423
1	-1.46317262	2.06277354	5.42785506
1	-0.16711115	2.72404562	4.38013066
6	-2.02918045	3.89406121	4.36900237
1	-2.15028313	4.39591171	5.33869489
1	-1.50906692	4.58523292	3.68811918
6	-3.34785561	3.43239504	3.74777371
1	-3.89514417	4.23549781	3.23641707
1	-4.00815100	3.00390632	4.51856090
6	-2.87426056	2.35301305	2.78348974
1	-2.59138124	2.76835732	1.80213211
1	-3.60919758	1.54994838	2.62195252

```
128
Eu_thf_3_2_N2Ph2_2_AV11__Ci__wb97xd_Def2TZV_MWB53
63       0.46893805      0.11090084     -1.74543530
8       -1.09355151      0.32883627     -3.81262319
8        2.14191866      1.51954727     -3.14322687
8        1.77297586     -1.52949452     -3.31685031
7       -0.69790438     -1.66032311      0.11694180
7        0.77207989     -1.57684081      0.08434138
6       -1.31148827     -2.47465281     -0.79497179
6       -0.64824210     -3.36580993     -1.67851421
1        0.42590970     -3.45697006     -1.60319429
6       -1.36676325     -4.13089440     -2.59100358
1       -0.83200666     -4.81428888     -3.24169079
6       -2.75428916     -4.04990256     -2.67885739
1       -3.30216644     -4.65539566     -3.38711039
6       -3.42513829     -3.18083164     -1.80792433
1       -4.50501290     -3.10411677     -1.85414277
6       -2.73161202     -2.41063722     -0.89505683
1       -3.25119943     -1.72889874     -0.23253809
6        1.46814741     -2.51017920      0.80776067
6        2.86193378     -2.29671005      1.00051843
1        3.29872700     -1.40614330      0.56376105
6        3.63153175     -3.18022287      1.73381395
1        4.69052357     -2.98417540      1.85744410
6        3.06162285     -4.31308410      2.32756870
1        3.66740467     -5.00206181      2.89959531
6        1.69964867     -4.54151216      2.14291570
1        1.24165330     -5.42327727      2.57646099
6        0.91001233     -3.66982179      1.40096710
1       -0.13753350     -3.87896521      1.24462713
6       -2.09521041     -0.71442134     -4.06083443
1       -1.92869170     -1.51685905     -3.34777102
1       -1.96922835     -1.09466087     -5.07804763
6       -3.41546107      0.01623238     -3.88483096
1       -3.61792215      0.14245650     -2.82062143
1       -4.24891932     -0.51426063     -4.34139550
6       -3.13223709      1.37633234     -4.54839743
1       -3.74564859      2.17423666     -4.13806452
1       -3.31056886      1.31593797     -5.62337546
6       -1.64398808      1.62422382     -4.25594301
1       -1.08720579      1.94728012     -5.13605123
1       -1.49458761      2.33547441     -3.44661780
6        2.10153821      1.39002196     -4.60290579
1        1.23379168      0.77785465     -4.84064775
1        3.00223232      0.87164849     -4.93169735
6        2.00396968      2.82652263     -5.15727781
1        1.19762348      2.92654027     -5.88230859
1        2.93452645      3.11065279     -5.64997632
6        1.76964483      3.70043881     -3.90746115
1        2.17252959      4.70571953     -4.01819312
1        0.70599637      3.77501289     -3.67801809
6        2.46269526      2.90762245     -2.80854843
1        3.54785708      3.03931956     -2.82689623
1        2.07973489      3.07649271     -1.80627527
6        1.29407257     -2.37755871     -4.40760992
1        1.52537077     -1.88212972     -5.35332269
1        0.21695670     -2.48242838     -4.30006619
6        2.04811472     -3.69683036     -4.25154972
1        2.17644572     -4.21374799     -5.20105235
1        1.51212084     -4.35569310     -3.56781877
6        3.38235241     -3.25830475     -3.62503023
1        3.87841299     -4.06664957     -3.09145118
1        4.05913999     -2.88262411     -4.39459207
6        2.95511649     -2.13496601     -2.68405890
1        2.65762118     -2.50847619     -1.70393699
1        3.69940456     -1.34973898     -2.56445569
63      -0.44773493      0.14542728      1.80771655
8        1.11475463     -0.07250814      3.87490444
8       -2.12071554     -1.26321915      3.20550812
8       -1.75177274      1.78582264      3.37913156
7        0.71910751      1.91665124     -0.05466055
```

7	-0.75087676	1.83316894	-0.02206013
6	1.33269140	2.73098093	0.85725304
6	0.66944522	3.62213805	1.74079546
1	-0.40470657	3.71329819	1.66547554
6	1.38796637	4.38722253	2.65328483
1	0.85320978	5.07061700	3.30397204
6	2.77549229	4.30623069	2.74113864
1	3.32336957	4.91172379	3.44939164
6	3.44634141	3.43715977	1.87020558
1	4.52621602	3.36044489	1.91642402
6	2.75281514	2.66696535	0.95733808
1	3.27240255	1.98522686	0.29481934
6	-1.44694428	2.76650732	-0.74547942
6	-2.84073065	2.55303818	-0.93823718
1	-3.27752387	1.66247142	-0.50147980
6	-3.61032862	3.43655099	-1.67153270
1	-4.66932045	3.24050353	-1.79516285
6	-3.04041972	4.56941222	-2.26528745
1	-3.64620154	5.25838994	-2.83731406
6	-1.67844554	4.79784029	-2.08063445
1	-1.22045017	5.67960539	-2.51417974
6	-0.88880920	3.92614991	-1.33868585
1	0.15873663	4.13529333	-1.18234588
6	2.11641354	0.97074946	4.12311568
1	1.94989483	1.77318718	3.41005227
1	1.99043148	1.35098899	5.14032888
6	3.43666420	0.24009574	3.94711221
1	3.63912528	0.11387162	2.88290268
1	4.27012245	0.77058875	4.40367675
6	3.15344022	-1.12000421	4.61067868
1	3.76685172	-1.91790853	4.20034577
1	3.33177198	-1.05960984	5.68565671
6	1.66519120	-1.36789569	4.31822426
1	1.10840892	-1.69095199	5.19833248
1	1.51579073	-2.07914629	3.50889905
6	-2.08033509	-1.13369383	4.66518704
1	-1.21258855	-0.52152652	4.90292900
1	-2.98102919	-0.61532036	4.99397860
6	-1.98276656	-2.57019451	5.21955906
1	-1.17642036	-2.67021215	5.94458984
1	-2.91332333	-2.85432466	5.71225757
6	-1.74844170	-3.44411069	3.96974240
1	-2.15132647	-4.44939140	4.08047437
1	-0.68479325	-3.51868476	3.74029934
6	-2.44149214	-2.65129432	2.87082968
1	-3.52665396	-2.78299143	2.88917748
1	-2.05853177	-2.82016459	1.86855652
6	-1.27286944	2.63388684	4.46989117
1	-1.50416764	2.13845785	5.41560394
1	-0.19575357	2.73875650	4.36234744
6	-2.02691160	3.95315849	4.31383097
1	-2.15524260	4.47007611	5.26333360
1	-1.49091772	4.61202123	3.63010002
6	-3.36114929	3.51463288	3.68731148
1	-3.85720986	4.32297769	3.15373243
1	-4.03793686	3.13895223	4.45687332
6	-2.93391337	2.39129413	2.74634015
1	-2.63641806	2.76480432	1.76621824
1	-3.67820144	1.60606710	2.62673694

128

Eu_thf_3_2_N2Ph2_2_AV11__Ci__wb97xd_GD2_Def2SVP_MWB53

63	0.36661074	0.10675348	-1.76873291
8	-1.12429357	0.34321391	-3.91924876
8	2.07351686	1.50825601	-3.18753132
8	1.73301616	-1.52423257	-3.33384249
7	-0.66563162	-1.63668760	0.11811761
7	0.76085916	-1.56100614	0.07710899
6	-1.29073512	-2.42378899	-0.78655898
6	-0.64017149	-3.33115763	-1.67068428
1	0.43997257	-3.45557444	-1.58284044
6	-1.37037519	-4.08231961	-2.59073568
1	-0.83750081	-4.78150892	-3.24353655
6	-2.75724012	-3.97475983	-2.69015874
1	-3.31699821	-4.57264417	-3.41147987
6	-3.41544766	-3.09000383	-1.82162533
1	-4.50316169	-2.98677640	-1.87583516
6	-2.71230950	-2.33100169	-0.90211204
1	-3.23157496	-1.63200299	-0.24007532
6	1.45877471	-2.45688042	0.81729278
6	2.85539630	-2.23691132	1.01083611
1	3.30150247	-1.35335239	0.54416391
6	3.62524212	-3.10031720	1.77338310
1	4.69313423	-2.89420768	1.89484060
6	3.05736288	-4.21979470	2.39891493
1	3.66830749	-4.89634781	2.99933100
6	1.69370986	-4.45396613	2.21651721
1	1.22982750	-5.33091779	2.67856120
6	0.90197591	-3.60343623	1.44732482
1	-0.15401861	-3.82428654	1.29126721
6	-2.09439615	-0.68435183	-4.12891866
1	-1.90631744	-1.49723670	-3.41429119
1	-1.99609885	-1.08757511	-5.15385418
6	-3.42025262	0.02453360	-3.92868662
1	-3.60016923	0.16144547	-2.85078569
1	-4.26833766	-0.52765995	-4.35540128
6	-3.15291890	1.36814635	-4.61165425
1	-3.77063173	2.18504849	-4.21830515
1	-3.33494545	1.28778188	-5.69470860
6	-1.66557684	1.61080890	-4.32697359
1	-1.11589934	1.96689070	-5.21355791
1	-1.51341355	2.32838934	-3.50705141
6	2.06547961	1.38080588	-4.60808084
1	1.18704307	0.77833663	-4.88171612
1	2.96820452	0.83803721	-4.93469805
6	2.01412756	2.81548071	-5.16935312
1	1.21491660	2.94123637	-5.91299752
1	2.96401233	3.07106246	-5.66230559
6	1.80243351	3.68363918	-3.92184759
1	2.24751305	4.68417563	-4.01001887
1	0.73129909	3.80250203	-3.69682442
6	2.44806848	2.83786717	-2.83737442
1	3.55021715	2.93086640	-2.84771365
1	2.07759060	3.02172960	-1.82060017
6	1.27888850	-2.33532246	-4.41297643
1	1.49229618	-1.81692995	-5.36523183
1	0.19225030	-2.47316093	-4.31914488
6	2.05551082	-3.64120160	-4.29306836
1	2.17895282	-4.15018462	-5.25874486
1	1.53469610	-4.32764925	-3.60790119
6	3.37255534	-3.17340459	-3.67304165
1	3.91980843	-3.97201918	-3.15467391
1	4.03369991	-2.75009698	-4.44594443
6	2.89622324	-2.08732794	-2.71746023
1	2.61467876	-2.49591327	-1.73294399
1	3.62928119	-1.28117936	-2.56266352
63	-0.34540761	0.14957465	1.83101416
8	1.14549670	-0.08688578	3.98153001
8	-2.05231373	-1.25192789	3.24981257
8	-1.71181304	1.78056069	3.39612374
7	0.68683475	1.89301573	-0.05583636

7	-0.73965603	1.81733426	-0.01482774
6	1.31193825	2.68011711	0.84884023
6	0.66137461	3.58748575	1.73296553
1	-0.41876944	3.71190257	1.64512169
6	1.39157832	4.33864773	2.65301693
1	0.85870393	5.03783704	3.30581780
6	2.77844325	4.23108795	2.75243999
1	3.33820134	4.82897229	3.47376112
6	3.43665079	3.34633196	1.88390658
1	4.52436481	3.24310452	1.93811641
6	2.73351262	2.58732981	0.96439329
1	3.25277809	1.88833111	0.30235657
6	-1.43757158	2.71320854	-0.75501153
6	-2.83419318	2.49323945	-0.94855486
1	-3.28029934	1.60968052	-0.48188266
6	-3.60403899	3.35664532	-1.71110185
1	-4.67193110	3.15053581	-1.83255935
6	-3.03615975	4.47612283	-2.33663368
1	-3.64710437	5.15267593	-2.93704975
6	-1.67250674	4.71029426	-2.15423596
1	-1.20862437	5.58724592	-2.61627995
6	-0.88077278	3.85976436	-1.38504357
1	0.17522173	4.08061466	-1.22898596
6	2.11559928	0.94067995	4.19119991
1	1.92752056	1.75356482	3.47657244
1	2.01730198	1.34390323	5.21613543
6	3.44145574	0.23179453	3.99096787
1	3.62137235	0.09488266	2.91306694
1	4.28954078	0.78398807	4.41768253
6	3.17412202	-1.11181823	4.67393550
1	3.79183486	-1.92872036	4.28058640
1	3.35614857	-1.03145375	5.75698985
6	1.68677997	-1.35448077	4.38925484
1	1.13710246	-1.71056257	5.27583916
1	1.53461668	-2.07206122	3.56933266
6	-2.04427649	-1.12447776	4.67036209
1	-1.16583994	-0.52200850	4.94399737
1	-2.94700140	-0.58170909	4.99697930
6	-1.99292444	-2.55915258	5.23163437
1	-1.19371347	-2.68490825	5.97527877
1	-2.94280920	-2.81473433	5.72458684
6	-1.78123039	-3.42731106	3.98412884
1	-2.22630993	-4.42784751	4.07230012
1	-0.71009597	-3.54617390	3.75910567
6	-2.42686536	-2.58153905	2.89965567
1	-3.52901402	-2.67453827	2.90999490
1	-2.05638747	-2.76540147	1.88288142
6	-1.25768537	2.59165058	4.47525768
1	-1.47109306	2.07325807	5.42751308
1	-0.17104717	2.72948906	4.38142613
6	-2.03430770	3.89752972	4.35534961
1	-2.15774969	4.40651274	5.32102611
1	-1.51349298	4.58397737	3.67018244
6	-3.35135221	3.42973271	3.73532290
1	-3.89860531	4.22834730	3.21695516
1	-4.01249678	3.00642510	4.50822568
6	-2.87502012	2.34365607	2.77974148
1	-2.59347563	2.75224140	1.79522524
1	-3.60807806	1.53750748	2.62494477

```
128
Eu_thf_3_2_N2Ph2_2_AV11__Ci__wb97xd_GD2_Def2TZV_MWB53
63       0.46375453      0.11054120     -1.74516349
8       -1.09634016      0.33023220     -3.81532815
8        2.13909787      1.51860747     -3.14002250
8        1.76701995     -1.52836880     -3.31869445
7       -0.68842805     -1.65907512      0.11474169
7        0.78181468     -1.57792884      0.08244175
6       -1.30357782     -2.47760289     -0.79269070
6       -0.64252503     -3.37739615     -1.66883728
1        0.43098418     -3.47344690     -1.59104378
6       -1.36266770     -4.14520360     -2.57780741
1       -0.82968250     -4.83529846     -3.22281378
6       -2.74965183     -4.05875524     -2.66889842
1       -3.29882894     -4.66639598     -3.37431440
6       -3.41842485     -3.18138248     -1.80483601
1       -4.49786678     -3.10033503     -1.85353911
6       -2.72316625     -2.40832410     -0.89558768
1       -3.24089516     -1.71987516     -0.23855933
6        1.47640006     -2.51045796      0.80807494
6        2.87041034     -2.29818275      1.00100317
1        3.30820502     -1.40907371      0.56233009
6        3.63884144     -3.18093670      1.73633958
1        4.69797360     -2.98569198      1.86001136
6        3.06756421     -4.31218105      2.33208769
1        3.67247959     -5.00075121      2.90551993
6        1.70544985     -4.53943914      2.14744381
1        1.24631375     -5.41989675      2.58244886
6        0.91688884     -3.66830505      1.40361022
1       -0.13072332     -3.87704752      1.24738559
6       -2.10266563     -0.70692510     -4.06899764
1       -1.94484568     -1.51056563     -3.35542324
1       -1.97393725     -1.08748018     -5.08573659
6       -3.41976977      0.03096249     -3.89895503
1       -3.62693842      0.15770131     -2.83572444
1       -4.25390509     -0.49433091     -4.36030719
6       -3.12563038      1.38992732     -4.55993859
1       -3.73654672      2.19094419     -4.15193614
1       -3.29884765      1.33130847     -5.63584186
6       -1.63752265      1.62922979     -4.25972153
1       -1.07447507      1.95043133     -5.13650335
1       -1.48837223      2.33854836     -3.44870566
6        2.10410623      1.38739125     -4.59962946
1        1.23945195      0.77183403     -4.83958260
1        3.00772886      0.87194282     -4.92502035
6        2.00383124      2.82309836     -5.15585539
1        1.19760575      2.92065504     -5.88133750
1        2.93398201      3.10843058     -5.64860789
6        1.76720622      3.69815149     -3.90730283
1        2.16926189      4.70371146     -4.01855606
1        0.70322670      3.77188385     -3.67907812
6        2.45963127      2.90707339     -2.80669038
1        3.54480276      3.03882408     -2.82466240
1        2.07649240      3.07753347     -1.80476981
6        1.28472208     -2.37966299     -4.40530703
1        1.50807520     -1.88438127     -5.35301739
1        0.20871576     -2.48899559     -4.29095033
6        2.04474485     -3.69600806     -4.25251465
1        2.17110727     -4.21225731     -5.20265492
1        1.51414007     -4.35704403     -3.56680276
6        3.37993290     -3.25265302     -3.63149092
1        3.88074233     -4.05904727     -3.09939201
1        4.05250937     -2.87512984     -4.40384648
6        2.95266641     -2.13011299     -2.68940238
1        2.65927674     -2.50381113     -1.70813358
1        3.69505773     -1.34265986     -2.57261801
63      -0.44255141      0.14578692      1.80744474
8        1.11754328     -0.07390407      3.87760940
8       -2.11789475     -1.26227935      3.20230375
8       -1.74581683      1.78469692      3.38097570
7        0.70963117      1.91540325     -0.05246044
```

7	-0.76061156	1.83425696	-0.02016050
6	1.32478095	2.73393101	0.85497195
6	0.66372816	3.63372427	1.73111853
1	-0.40978105	3.72977502	1.65332503
6	1.38387083	4.40153173	2.64008866
1	0.85088563	5.09162658	3.28509503
6	2.77085495	4.31508337	2.73117967
1	3.32003207	4.92272410	3.43659565
6	3.43962798	3.43771061	1.86711726
1	4.51906990	3.35666316	1.91582036
6	2.74436938	2.66465222	0.95786893
1	3.26209829	1.97620328	0.30084058
6	-1.45519693	2.76678608	-0.74579369
6	-2.84920722	2.55451087	-0.93872192
1	-3.28700190	1.66540184	-0.50004884
6	-3.61763832	3.43726482	-1.67405833
1	-4.67677048	3.24202011	-1.79773011
6	-3.04636109	4.56850918	-2.26980644
1	-3.65127646	5.25707934	-2.84323868
6	-1.68424672	4.79576727	-2.08516256
1	-1.22511063	5.67622488	-2.52016761
6	-0.89568572	3.92463317	-1.34132897
1	0.15192644	4.13337565	-1.18510434
6	2.12386876	0.96325323	4.13127889
1	1.96604880	1.76689375	3.41770449
1	1.99514037	1.34380831	5.14801784
6	3.44097290	0.22536563	3.96123628
1	3.64814154	0.09862681	2.89800569
1	4.27510822	0.75065904	4.42258844
6	3.14683351	-1.13359919	4.62221984
1	3.75774984	-1.93461607	4.21421739
1	3.32005077	-1.07498035	5.69812311
6	1.65872577	-1.37290167	4.32200278
1	1.09567820	-1.69410321	5.19878460
1	1.50957535	-2.08222024	3.51098691
6	-2.08290311	-1.13106312	4.66191071
1	-1.21824883	-0.51550590	4.90186385
1	-2.98652574	-0.61561470	4.98730160
6	-1.98262811	-2.56677024	5.21813664
1	-1.17640262	-2.66432692	5.94361875
1	-2.91277888	-2.85210245	5.71088914
6	-1.74600310	-3.44182337	3.96958408
1	-2.14805876	-4.44738334	4.08083731
1	-0.68202357	-3.51555572	3.74135937
6	-2.43842815	-2.65074527	2.86897163
1	-3.52359963	-2.78249596	2.88694365
1	-2.05528928	-2.82120534	1.86705106
6	-1.26351895	2.63599112	4.46758828
1	-1.48687207	2.14070940	5.41529864
1	-0.18751263	2.74532371	4.35323158
6	-2.02354173	3.95233618	4.31479590
1	-2.14990414	4.46858544	5.26493617
1	-1.49293694	4.61337215	3.62908401
6	-3.35872977	3.50898114	3.69377217
1	-3.85953921	4.31537539	3.16167326
1	-4.03130625	3.13145797	4.46612773
6	-2.93146329	2.38644112	2.75168363
1	-2.63807361	2.76013925	1.77041483
1	-3.67385461	1.59898799	2.63489926

Table S3. Differences from experiment (Å) for symmetry-unique Eu-R (R = Eu/O/N/Cp) interatomic distances across theoretical methods. Experimental values are provided at table bottom.

**Method**	**Eu-Eu**	**Eu-O1**	**Eu-O2**	**Eu-O3**	**Eu-N1**	**Eu-N2**	**Eu-N3**	**Eu-N4**	**Eu-C1**	**Eu-C2**
B3LYP/Def2-SVP	0.037	0.098	0.091	0.086	0.100	0.074	0.053	0.048	0.103	0.121
B3LYP/Def2-TZV	0.046	0.083	0.055	0.065	0.148	0.073	0.055	0.095	0.202	0.201
B3LYP-GD3(BJ)/Def2-SVP	0.024	0.032	0.009	-0.007	0.115	0.053	0.023	0.029	0.065	0.094
B3LYP-GD3(BJ)/Def2-TZV	0.016	0.013	-0.017	-0.027	0.144	0.049	0.023	0.057	0.133	0.159
CAM-B3LYP/Def2-SVP	0.000	0.070	0.047	0.041	0.072	0.065	0.043	0.031	0.052	0.087
CAM-B3LYP/Def2-TZV	0.000	0.049	0.010	0.017	0.116	0.061	0.044	0.071	0.155	0.167
CAM-B3LYP-GD3(BJ)/Def2-SVP	-0.015	0.031	0.003	-0.008	0.078	0.053	0.023	0.015	0.029	0.072
CAM-B3LYP-GD3(BJ)/Def2-TZV	-0.022	0.011	-0.027	-0.032	0.115	0.047	0.022	0.046	0.117	0.148
LC-ωHPBE/Def2-SVP	-0.006	0.075	0.058	0.059	0.051	0.056	0.038	0.000	0.036	0.021
LC-ωHPBE/Def2-TZV	-0.011	0.045	0.015	-0.009	0.028	0.046	0.028	-0.003	0.034	-0.050
LC-ωHPBE-GD3(BJ)/Def2-SVP	-0.007	0.052	0.018	-0.014	0.033	0.046	0.030	0.001	0.037	-0.041
LC-ωHPBE-GD3(BJ)/Def2-TZV	-0.036	0.024	-0.003	-0.030	0.049	0.037	0.022	0.028	0.110	0.043
ωB97XD/Def2-SVP	0.047	0.036	0.009	-0.018	0.092	0.062	0.027	0.022	-0.014	0.053
ωB97XD/Def2-TZV	0.047	0.009	-0.021	-0.039	0.151	0.051	0.018	0.049	0.091	0.141
ωB97XD-GD2/Def2-SVP	0.048	0.036	0.008	-0.017	0.097	0.062	0.027	0.012	-0.009	0.045
ωB97XD-GD2/Def2-TZV	0.044	0.010	-0.022	-0.039	0.143	0.052	0.018	0.053	0.087	0.143
Experiment	3.622	2.592	2.617	2.658	2.671	2.456	2.469	2.676	3.189	3.283

Table S4. RMSD, NPA natural charges, N=N stretching modes, gas-phase frequencies (cm^{-1}) for cis-azobenzene, and average time-per-SCF cycle (SCF, min:sec) on a 16-core 3.3 GHz computer.

**Method**	**RMSD**	**NPA_{Eu}**	**NPA_{N1}**	**NPA_{N2}**	**Ng**	**Nu**	**N-cis**	**% vs. Exp.**	**N NPA**	**SCF**
B3LYP/Def2-SVP	0.284	1.572	-0.676	-0.690	1003	1004	1679	11.1	-0.142	10:36
B3LYP/Def2-TZV	0.358	1.238	-0.615	-0.621	1009	1010	1472	-2.6	-0.106	25:06
B3LYP-GD3(BJ)/Def2-SVP	0.305	1.520	-0.664	-0.680	1012	1013	1679	11.1	-0.143	11:22
B3LYP-GD3(BJ)/Def2-TZV	0.309	1.211	-0.597	-0.594	1016	1016	1471	-2.6	-0.106	25:57
CAM-B3L/YP/Def2-SVP	0.288	1.498	-0.699	-0.710	1032	1033	1751	15.9	-0.139	14:47
CAM-B3LYP/Def2-TZV	0.312	1.247	-0.616	-0.626	1029	1030	1566	3.6	-0.104	37:24
CAM-B3LYP-GD3(BJ)/Def2-SVP	0.285	1.476	-0.680	-0.693	1036	1038	1750	15.8	-0.139	20:02
CAM-B3LYP-GD3(BJ)/Def2-TZV	0.296	1.236	-0.604	-0.616	1032	1033	1564	3.5	-0.104	32:32
LC-ωHPBE/Def2-SVP	0.231	1.513	-0.690	-0.708	1071	1073	1815	20.1	-0.133	22:02
					1060	1062				
LC-ωHPBE/Def2-TZV	0.151	1.287	-0.619	-0.634	997	1002	1642	8.6	-0.097	48:41
					1042	1043				
LC-ωHPBE-GD3(BJ)/Def2-SVP	0.165	1.493	-0.688	-0.691	1063	1064	1811	19.8	-0.133	18:21
					1074	1076				
LC-ωHPBE-GD3(BJ)/Def2-TZV	0.178	1.239	-0.614	-0.613	1002	1005	1639	8.4	-0.097	39:22
					1046	1047				
ωB97XD/Def2-SVP	0.319	1.494	-0.679	-0.692	1039	1041	1740	15.2	-0.140	18:39
ωB97XD/Def2-TZV	0.325	1.224	-0.599	-0.616	1030	1030	1559	3.1	-0.101	39:42
ωB97XD-GD2/Def2-SVP	0.318	1.497	-0.678	-0.690	1039	1041	1740	15.2	-0.140	16:42
ωB97XD-GD2/Def2-TZV	0.324	1.224	-0.598	-0.617	1030	1030	1559	3.1	-0.101	36:17
