# Supplementary material for: Azobenzene as an Effective Ligand in Europium Chemistry—A Synthetic and Theoretical Study
Source: Molecules. 2024 Nov 2;29(21):5187. doi: 10.3390/molecules29215187 (PMC11547820; doi:10.3390/molecules29215187)

checkCIF/PLATON report

Structure factors have been supplied for datablock(s) at-av111_a

THIS REPORT IS FOR GUIDANCE ONLY. IF USED AS PART OF A REVIEW PROCEDURE FOR PUBLICATION, IT SHOULD NOT REPLACE THE EXPERTISE OF AN EXPERIENCED CRYSTALLOGRAPHIC REFEREE.

No syntax errors found. CIF dictionary Interpreting this report

Datablock: at-av111_a

```
Bond precision:   C-C = 0.0046 A           Wavelength=0.71073

Cell:             a=10.9632(7)     b=13.5363(9)       c=15.7256(10)
                  alpha=90         beta=91.565(1)     gamma=90
Temperature:      100 K

                 Calculated                     Reported
Volume           2332.8(3)                      2332.8(3)
Space group      P 21/n                         P 1 21/n 1
Hall group       -P 2yn                         -P 2yn
Moiety formula   C48 H68 Eu2 N4 O6              C48 H68 Eu2 N4 O6
Sum formula      C48 H68 Eu2 N4 O6              C48 H68 Eu2 N4 O6
Mr               1101.00                        1100.98
Dx,g cm-3        1.567                          1.567
Z                2                              2
Mu (mm-1)        2.715                          2.715
F000             1116.0                         1116.0
F000'            1115.72
h,k,lmax         13,16,18                       13,16,18
Nref             4112                           4110
Tmin,Tmax        0.391,0.494                    0.601,0.746
Tmin'            0.362

Correction method= # Reported T Limits: Tmin=0.601 Tmax=0.746
AbsCorr = MULTI-SCAN

Data completeness= 1.000           Theta(max)= 24.997

R(reflections)= 0.0240( 3803)                          wR2(reflections)=
                                                       0.0606( 4110)
S = 1.106                  Npar= 271
```

The following ALERTS were generated. Each ALERT has the format

test-name_ALERT_alert-type_alert-level.

Click on the hyperlinks for more details of the test.

Alert level B

```
PLAT094_ALERT_2_B Ratio of Maximum / Minimum Residual Density ....       4.76 Report
```

Author Response: Residual electron density peaks probably arise from strong absorption effects that were not fully corrected for in absorption correction of heavy Eu metal.

Alert level C

```
PLAT232_ALERT_2_C Hirshfeld Test Diff (M-X)  Eu1      --N1_a     .         5.3 s.u.
PLAT911_ALERT_3_C Missing FCF Refl Between Thmin & STh/L=    0.595           2 Report
                 8  2  0,   1  3 18,
PLAT971_ALERT_2_C Check Calcd Resid. Dens.  0.90Ang From Eu1              1.61 eA-3
```

Alert level G

```
PLAT909_ALERT_3_G Percentage of I>2sig(I) Data at Theta(Max) Still        87% Note
PLAT933_ALERT_2_G Number of HKL-OMIT Records in Embedded .res File          2 Note
                 1  3 18,   8  2  0,
PLAT941_ALERT_3_G Average HKL Measurement Multiplicity ..........         3.9 Low
PLAT967_ALERT_5_G Note: Two-Theta Cutoff Value in Embedded .res ..       50.0 Degree
PLAT969_ALERT_5_G The 'Henn et al.' R-Factor-gap value ..........       3.00 Note
               Predicted wR2: Based on SigI**2  2.02 or SHELX Weight  5.66
PLAT978_ALERT_2_G Number C-C Bonds with Positive Residual Density.          8 Info
```

0 **ALERT level A** = Most likely a serious problem - resolve or explain
1 **ALERT level B** = A potentially serious problem, consider carefully
3 **ALERT level C** = Check. Ensure it is not caused by an omission or oversight
6 **ALERT level G** = General information/check it is not something unexpected

0 ALERT type 1 CIF construction/syntax error, inconsistent or missing data
5 ALERT type 2 Indicator that the structure model may be wrong or deficient
3 ALERT type 3 Indicator that the structure quality may be low
0 ALERT type 4 Improvement, methodology, query or suggestion
2 ALERT type 5 Informative message, check

It is advisable to attempt to resolve as many as possible of the alerts in all categories. Often the minor alerts point to easily fixed oversights, errors and omissions in your CIF or refinement strategy, so attention to these fine details can be worthwhile. In order to resolve some of the more serious problems it may be necessary to carry out additional measurements or structure refinements. However, the purpose of your study may justify the reported deviations and the more serious of these should normally be commented upon in the discussion or experimental section of a paper or in the "special_details" fields of the CIF. checkCIF was carefully designed to identify outliers and unusual parameters, but every test has its limitations and alerts that are not important in a particular case may appear. Conversely, the absence of alerts does not guarantee there are no aspects of the results needing attention. It is up to the individual to critically assess their own results and, if necessary, seek expert advice.

Publication of your CIF in IUCr journals

A basic structural check has been run on your CIF. These basic checks will be run on all CIFs submitted for publication in IUCr journals (*Acta Crystallographica*, *Journal of Applied Crystallography*, *Journal of Synchrotron Radiation*); however, if you intend to submit to *Acta Crystallographica Section C* or *E* or *IUCrData*, you should make sure that full publication checks are run on the final version of your CIF prior to submission.

Publication of your CIF in other journals

Please refer to the *Notes for Authors* of the relevant journal for any special instructions relating to CIF submission.

PLATON version of 06/01/2024; check.def file version of 05/01/2024

Datablock at-av111_a - ellipsoid plot

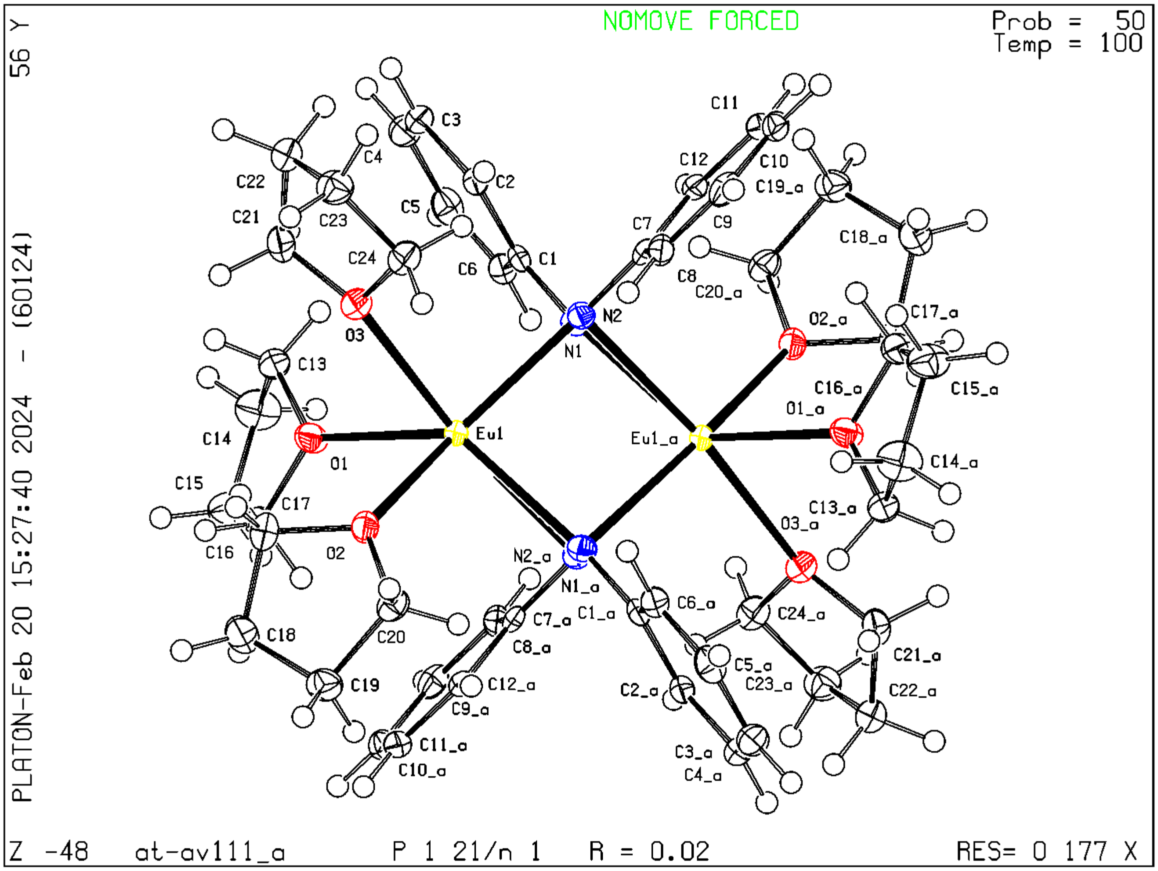

Supplement: Supplementary file 1 [file Eu_AzoTHF_Checkcif.pdf › Eu_AzoTHF_Checkcif.pdf]
